# Health-related quality of life in hemoglobinopathies: A systematic review from a global perspective

**DOI:** 10.3389/fped.2022.886674

**Published:** 2022-08-25

**Authors:** Francesca Rodigari, Giorgia Brugnera, Raffaella Colombatti

**Affiliations:** Department of Woman’s and Child’s Health, University of Padova, Padua, Italy

**Keywords:** sickle cell disease, thalassemia, hemoglobinopathies, health-related quality of life, PROMIS, SF-36, Peds-QOL

## Abstract

**Background:**

Sickle cell disease (SCD) and thalassemia are inherited blood disorders, which can lead to life-threatening events and chronic organ damage. Recent advances in treatments have increased life expectancy, and hemoglobinopathies have become chronic illnesses with social and emotional impairments. Thus, health-related quality of life (HRQOL) assessment has a fundamental role in disease management and treatment, and generic and disease-specific questionnaires are reliable and validated measures to estimate disease burden. The heterogeneous distribution of treatment opportunities worldwide influences physical, social, and emotional disease perception.

**Objectives:**

To review publications concerning HRQOL for SCD and thalassemia in different areas of the world in order to gather a global perspective of questionnaires used and outcomes evaluated.

**Methods:**

A systematic review of the literature was conducted according to the Preferred Reporting Items for Systematic Reviews and Meta-Analyses (PRISMA) guidelines. The Medline databases were searched on 29 September 2021. Inclusion criteria were as follows: (1) studies of HRQOL assessment in SCD and thalassemia patients by using the PROMIS, the SF-36, the SCSES, the PedsQL-SCD, the PedsQOL generic core scale, the ASCQ-Me, and the TranQoL; and (2) every article type, including non-English studies. We excluded studies that were not limited to SCD or thalassemia and studies that were not specific to hemoglobinopathies, and not consistent with the topic of HRQOL assessment. We did not include the gray literature. A total of 102 out of 124 articles from PubMed, Cochrane Library, and Google Scholar were eligible for inclusion (66 SCD articles and 36 thalassemia articles). The quality of studies was assessed through Critical Appraisal tools for use in JBI Systematic Reviews. Data extraction was conducted using a standardized data collection form (authors, year and country of publication, study design, age and number of patients, HRQOL questionnaires, questionnaire language, and clinical outcomes).

**Results:**

The evaluation of HRQOL was conducted on all continents, but differences in the worldwide frequency of HRQOL assessment were observed. HRQOL of SCD patients was less investigated in Europe. HRQOL of thalassemia patients was less investigated in South-East Asia and Africa. Generic HRQOL questionnaires (PROMIS, SF-36, and PedsQL) were frequently adopted, while disease-specific ones (ASCQ-Me, SCSES for SCD, and TranQoL for thalassemia) were less used. Translation into local languages has been often performed.

**Conclusion:**

Health-related quality of life is a complex outcome that has been increasingly incorporated in clinical research and clinical practice worldwide, although with regional differences. Disease-specific outcomes (pain for SCD and transfusion burden for thalassemia) and healthcare system characteristics, particularly in low-income countries, have an impact on HRQOL and should be considered in healthcare plans.

## Introduction

Sickle cell disease (SCD) and thalassemia are the most common monogenic diseases worldwide with an estimated incidence of 300–400,000 new children born per year (1). During the last 30 years, the improved comprehensive care coupled with the widespread use of disease-modifying treatments like hydroxyurea (HU) for SCD or new drugs, such as iron chelators for thalassemia, has increased the life expectancy of patients (2). Hemoglobinopathies have thus become chronic illnesses where social and emotional aspects play a fundamental role in disease management and adherence to therapy (3, 4). The evaluation of disease impact on physical and mental health, social life, work, and school is a clinical challenge that can help to assess unmet needs, adherence to therapy, and efficacy of new treatments (3, 5).

Individuals affected by hemoglobinopathies experience several disease-related acute and chronic complications across their lifespan, which decrease their health-related quality of life (HRQOL) (5, 6). HRQOL is a complex outcome that reflects how chronic disease is experienced by a patient and his/her family. For individuals with hemoglobinopathies, HRQOL assessment is recognized as an integral part of health research and has been recently incorporated into clinical care (7, 8).

Pain is a significant predictor of poor HRQOL in SCD: patients undergo frequent hospitalizations due to painful vaso-occlusive crises that affect physical and psychological functioning. Fatigue is an independent predictor of low HRQOL that has been associated with lower executive functioning and working memory of affected people (5, 8–10). Concurrently, regular blood transfusions and disease complications in beta-thalassemia patients affect HRQOL, and iron overload, comorbidities, and fatigue are the most represented etiologies of impaired life functioning (11–13). Furthermore, patients can encounter stigma for other reasons, such as race, disability, socioeconomic status, and delayed growth and puberty (3–14). The heterogeneous distribution of treatment opportunities and multidisciplinary management worldwide can also influence HRQOL perception in different settings (3, 4).

Patient-reported outcomes have emerged as a critical tool to measure SCD disease severity and response to treatment, facilitating a patient-centered approach (15). HRQOL assessment is based on the use of both generic and disease-specific measures of different quality of life domains, mainly through the administration of questionnaires. The most common generic health-related questionnaires are The Short Form (36) Health Survey (SF-36), the Patient-Reported Outcomes Measurement Information System (PROMIS), and the Pediatric Quality of Life Inventory (PedsQL).

**FIGURE 1:**
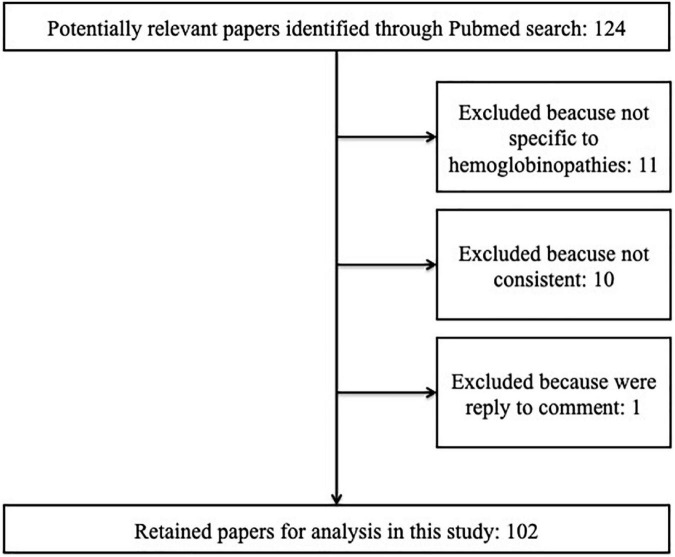
Review of literature and identification of studies.

The SF-36 (Supplementary material 1–Figure 1) is a 36-item patient-reported questionnaire on health status, which is widely used to assess chronic disease burden and the cost-effectiveness of treatments. It consists of eight sections of questions about, respectively, vitality, physical functioning, bodily pain, general health perceptions, physical role functioning, emotional role functioning, social role functioning, and mental health. Scores from each section are transformed into a 0–100 scale where a lower score indicates more disability and a higher score less disability. In addition, the eight domains could be summarized into two health components: the physical component score (PCS) and the mental component score (MCS) (16).

The PROMIS (Supplementary material 1–Figure 2) scale provides self-reported health measures in the domains of physical health, mental health, and social health. Under each main domain, there are sub-domains associated with symptoms, function, affect, behavior, cognition, relationships, or function (17–20).

The PedsQL (Supplementary material 1–Figure 3) scale is a brief generic assessment instrument that measures patients’ and parents’ perceptions of HRQOL in pediatric patients with chronic diseases. It consists of a 15-item core measure of global HRQOL and eight supplemental modules assessing specific symptoms or treatment domains (21). Some disease-specific modules for the PedsQL have been developed to assess particular health issues; for SCD, the PedsQL-SCD Module has been developed, which demonstrated acceptable measurement properties for pediatric patient self-report and parent proxy report (22–24).

Disease-specific questionnaires developed to assess HRQOL of SCD patients are the Sickle Cell Self-Efficacy Scale (SCSES) and the Adult Sickle Cell Quality of Life Measurement Information System (ASCQ-Me).

The SCSES (Supplementary material 1–Figure 4) is a nine-item disease-specific instrument measuring self-efficacy for managing sickle cell symptomatology in adults (25).

The ASCQ-Me (Supplementary material 1–Figure 5) is an outcome measurement system that assesses the impact of SCD on adult patients. It includes questions on seven main topics: emotional impact, pain episodes, pain impact, SCD medical history, sleep impact, social functioning, and stiffness impact (7, 26, 27). ASCQ-Me is a one-time tool that evaluates both acute and chronic pain; currently, there is no evidence to support its responsiveness to longitudinal changes over time (27).

For thalassemia patients, the Transfusion-dependent HRQOL questionnaire (TranQoL) has been developed, which is a disease-specific HRQOL measure for children and adults with thalassemia major. The included questions are grouped into four domains: physical health, emotional health, family functioning, and school and career functioning (28).

While the above-mentioned questionnaires have been used in various settings, a review of their application from a global perspective is lacking.

Considering the increased interest in documenting HRQOL in SCD and thalassemia, in this review, we aim to present HRQOL evaluation tools utilized in different countries and the results of the investigations worldwide. The objective is to find areas of potential improvement in the healthcare management of people with hemoglobin disorders.

## Materials and methods

We conducted a systematic review following the Preferred Reporting Items for Systematic Reviews and Meta-Analyses (PRISMA) guidelines (29). A literature search was conducted on September 29, 2021 in the databases of PubMed, Cochrane Library, and Google Scholar. The search was conducted using the following keywords: (PROMIS AND sickle cell disease) OR (PROMIS AND thalassemia) OR (SF-36 AND sickle cell disease) OR (SF-36 AND thalassemia) OR (SCSES AND sickle cell disease) OR (PedsQL-SCD AND sickle cell disease) OR (TranQoL AND thalassemia) OR (PedsQOL generic core scale AND thalassemia) OR (PedsQOL generic core scale AND sickle cell disease) OR (ASCQ-Me AND sickle cell disease).

We identified 124 papers published between 2000 and 2021 (Figure 1). The studies selected for our systematic review met the following criteria: (1) they investigated HRQOL assessment in SCD and thalassemia patients by using the PROMIS, the SF-36, the SCSES, the PedsQL-SCD, the PedsQOL generic core scale, the ASCQ-Me, and the TranQoL; (2) every article type was accepted, including non-English studies. Observational studies, randomized clinical trials, quasi-experimental studies, systematic and narrative reviews, qualitative studies, and case reports were found.

We excluded studies that were not limited to SCD or thalassemia and studies that were not specific to hemoglobinopathies (11) or not consistent with the topic of HRQOL assessment (11). We did not include the gray literature. Finally, 102 articles were selected and introduced for the quality assessment phase (66 papers on SCD and 36 on thalassemia) (Figure 1). The quality of studies was assessed through Critical Appraisal tools for use in JBI Systematic Reviews (Supplementary material 2). For each criterion of the JBI Critical Appraisal checklists, the included studies were classified by quality rating as good, fair, or poor by the reviewers. Two independent reviewers (FR and GB) performed the search, evaluation, and data extraction. The agreement between data extraction was discussed and supervised by a third person (RC).

Data extraction was conducted using a standardized data collection form, which included information about authors, year and country of publication, study design, age and number of patients, HRQOL questionnaires, questionnaire language, and clinical outcomes (Supplementary material 3).

## Results

### Sickle cell disease

We identified 66 articles on HRQOL in SCD patients, conducted in the Middle East (4 articles), Latin America and the Caribbean (14 articles), Africa (4 articles), the United States (44 articles), and Europe (1 article). Supplementary material 3 (Tables 1.1 and 1.2) summarize the characteristics of the SCD studies that have been selected. The SF-36 was the most common questionnaire adopted (26/66, 39%), followed by the PROMIS (19/66, 29%) and the PedsQL (15/66, 23%). ASCQ-Me was used in eight studies (12%) and SCSES in two studies (3%). Several studies adopted more questionnaires at the same time: one study adopted SF-36 and PedsQL, one PedsQL and PROMIS, two ASCQ-Me and PROMIS, and one ASCQ-Me and SF-36. We found one study that adopted a different questionnaire, the WHOQOL-Bref, combined with SF-36. One study was a review on the use of the patient-reported outcomes questionnaire (15). In some studies, questionnaires have been translated into local languages to perform the research, with an explicit mention in the Section “Materials and methods” (13/66, 20%).

Twenty-seven studies (41%) have been conducted mainly on the adult population (age > 18 years). Twenty-one studies (32%) included children and adolescents (age < 18 years), nine studies (14%) adolescents and adults (age > 14 years), six studies only children (age ≤ 14 years), and two studies children, adolescents, and adults. No studies included only the adolescent population (age 14–18 years).

#### Risk of bias assessment

Our review included 55 observational studies (83%), 1 randomized clinical trial (1,5%), 3 systematic reviews (4,5%), 1 narrative review (1,5%), 3 quasi-experimental studies (4,5%), 1 case report (1,5%), and 2 qualitative studies. It was not possible to assess the narrative review’s risk of bias through the JBI Critical Appraisal checklist, thus it was considered as “poor” due to the intrinsic limitations of the study type. The overall risk of bias assessment was low (Supplementary material 2–Table 1).

##### Risk of bias assessment for observational study

Critical Appraisal checklist for observational studies and related risk of bias assessment results are available in Supplementary material 2–Figures 1, 2 and Table 1. Among the 51 cross-sectional observational studies (51/55, 93%), 8 were rated “fair” and 43 were rated “good.” Prospective studies were four (4/55, 7%), and all were rated “good.”

##### Risk of bias assessment for clinical trials

Critical Appraisal checklist for randomized clinical trials and related risk of bias assessment results are available in Supplementary material 2 (Figure 3 and Table 1). The RCT study was rated “good.”

##### Risk of bias assessment for quasi-experimental study

The risk of bias assessment for quasi-experimental studies is available in Supplementary material 2 (Figure 4 and Table 1) and was rated “good.”

##### Risk of bias assessment for systematic reviews

A Critical Appraisal checklist for systematic review and related risk of bias assessment results are available in Supplementary material 2 (Figure 5 and Table 1). Three systematic reviews were included; among them, two were rated “good” and one was rated “poor.”

##### Risk of bias assessment for qualitative research

A Critical Appraisal checklist for qualitative studies and related risk of bias assessment results are available in Supplementary material 2 (Figure 6 and Table 1). Two qualitative studies were included, and both were rated “good.”

##### Risk of bias assessment for case report

A Critical Appraisal checklist for case reports and related risk of bias assessment results are available in Supplementary material 2 (Figure 7 and Table 1).

The case report was rated “good.”

#### Middle East

We found four articles on HRQOL in Middle East countries (two in Saudi Arabia, one in Iran, and one in Oman). Three studies used the SF-36 questionnaire, and one study adopted the PedsQL. Research on HRQOL of patients with SCD in Saudi Arabia explored factors associated with better or worse outcomes and showed that individuals with SCD exhibit a poor general HRQOL. In a large study conducted among 629 adult Saudi patients, it has been reported that fever, skin redness, and swelling were associated with poorer quality of life in multiple domains of the SF-36. Patients with a university degree and who were employed had better scores. The HRQOL of patients with a history of blood transfusion was found to be poorer, whereas regular exercise tended to improve vitality, social function, general health, and reduce pain (30). These results have been confirmed by a recent study conducted on 107 adult patients in the southern region of Saudi Arabia, in which, in addition, family support was shown to increase bodily pain scores and general health status (31).

Studies conducted in Oman and Iran focused on interventions designed to teach patients and caregivers how to manage and control the disease and the effect of such interventions on HRQOL (32, 33). The 5A self-management model (Assess, Advise, Agree, Assistant, and Arrange) has been demonstrated to improve the quality of life of patients, as assessed through the Persian version of the SF-36 questionnaire administered before and after the administration of the 5A model (32). Educational sessions on basic understanding of SCD and homecare management of signs and symptoms can significantly improve the HRQOL PedsQL score (33).

#### Latin America and the Caribbean

Studies on HRQOL in Latin America and the Caribbean are mainly conducted in Brazil (eight studies) and Jamaica (six studies). Twelve studies adopted the SF-36, and three studies the PedsQL (in one case combined with SF-36). Seven studies declared that the questionnaire adopted was translated into the local language (Portuguese).

In Jamaica, the SF-36 appears to be the most used HRQOL questionnaire, which was validated in different SCD populations (34, 35). However, it has been shown that the WHOQOL-Bref instrument, a 26-item instrument that measures broader and subjective domains (physical health, psychological health, social relationships, and environmental health), is a good instrument in determining HRQOL in Jamaican patients. Its utility is comparable to that of the SF-36 and Flanagan’s quality of life scale, a generic scale adapted for use among persons with chronic diseases (36).

Brazilian patients and their parents have shown low scores on the HRQOL assessment with SF-36 (37). HRQOL perception is influenced by several factors, including psychological aspects. In particular, Brazilian SCD patients who had already perceived any kind of prejudice had statistically significantly worse HRQOL (38). Additionally, the HRQOL of Brazilian children seems to vary according to the health center they visit for treatment, as evidenced by the administration of the PedsQL-SCD module in 412 children from six different Brazilian health centers (39). Jamaican studies regarding parents’ and adolescents’ perception of HRQOL exhibited overall general agreement but differences were observed on subjective domains, as assessed with the PedsQLSCD module. The HRQOL of girls is generally overestimated by the parental proxy (40, 41).

Many Latin American and Caribbean states are developing countries where there are rural–urban differences in health services. Rural–urban differences in the HRQOL of Jamaican patients have been investigated through SF-36. Employed Jamaican patients and those in higher-level occupations, such as professionals, showed lower limitations in their role functioning. Interestingly, rural patients assessed higher HRQOL than urban patients, despite having less access to healthcare (42).

In Brazil, the impact of SCD on pain, muscle function, and working capacity has been widely investigated. Pain in different regions of the body influences different aspects of the HRQOL assessed through the SF-36. Pain in the upper limbs reduced physical functioning, vitality, role-physical, and mental health, and was also associated with race (blacks had more pain) and education (low schooling was associated with pain). Pain in the spine and the lower limbs contributed to a reduction in the scores of physical functioning, role-physical, general health, mental health, and bodily pain scores (43). Adult patients show muscle dysfunction, in particular the endurance of the knee flexor muscles, with an impact on HRQOL. The use of hydroxyurea is associated with less muscle dysfunction and better HRQOL scores (44). The disease interferes with the working capacity of Brazilian patients, who mostly have low incomes and impaired access to healthcare services, and significantly impacts their HRQOL (45).

Physical rehabilitation appears to potentially increase the functional capacity of patients, avoiding deconditioning and exercise intolerance. A study conducted in Rio de Janeiro reported the positive results of a home-based, manually guided physical rehabilitation program, with an increase in functional capacity, muscle strength, and HRQOL assessed with the SF-36 (46). Aquatic rehabilitation programs can improve HRQOL, significantly decreasing pain and increasing the strength of respiratory muscles (47).

A Jamaican study investigated the relationship between locus of control (LOC) and HRQOL assessed through SF-36. The perception of life as being determined by oneself behavior, a concept known as internal locus of control, is associated with high HRQOL, while the perception of life as being determined by external factors, known as external LOC, is associated with depression (48).

#### Africa

We found four studies on HRQOL in African SCD patients. Two studies used the SF-36, one study the PedsQL, and one study the SCSES.

In SCD patients living in Cameroon, the most frequently reported chronic complications are nocturnal enuresis, chronic leg ulcers, osteomyelitis, and priapism, followed by stroke and avascular necrosis. A globally low HRQOL was reported, which worsened by age, urban residence, and history of stroke (49).

The administration of the PedsQL tool to a population of Mozambican pediatric patients and caregivers showed the lower quality of life in emotional and communication domains than in the pain-related domain (50).

In Nigerian adult patients, hopelessness appears to be influenced by hemoglobin concentration and psychological variables, such as resilience and depression, assessed through a specific scale for hopelessness, anxiety, and resilience and the Sickle Cell Self-Efficacy Scale (SCSES) (51).

The HRQOL measurement through the utility approach, a preference-based approach that focuses on individual preferences about experiencing a given health state, showed that the utility score in Nigerian SCD patients is low, indicating the strong impact of the disease on HRQOL, with limitations in everyday life. The score increased with the level of education but decreased with age, anxiety, frequency of pain episodes, and the number of comorbidities (52).

#### United States

The majority of studies on HRQOL in SCD patients have been conducted in the United States (42/66, 64%).

The assessment of HRQOL through ASCQ-Me and PROMIS questionnaires allowed researchers to associate outcomes to demographic and clinical characteristics, which is fundamental to understand new targets for interventions and the relative economic burden (53, 54). In addition, some studies have been conducted on the internal validity of HRQOL questionnaires and their usefulness in the research context. For example, several PROMIS pediatric measures have been demonstrated to be responsive to changes in health status, particularly with the occurrence and resolution of acute VOC pain requiring hospitalization (55–57). Compared to PROMIS scores, most ASCQ-Me scores are better predictors of SCD disease severity (7, 26, 58, 59). However, differently from PROMIS, the use of ASCQ-Me has not been validated for longitudinal changes (19, 55, 56, 60).

A problem that emerged in a multicultural context like the United States is that HRQOL assessment tools must be adequate to the population language, for example, for Spanish speakers of the Latino minority. A Spanish translation of the PedsQL-SCD module has been demonstrated to be a valid instrument for the measurement of HRQOL in patients and their families (33).

The SCD patients show a worse HRQOL than the general population, and their SF-36 scores are similar to patients with other chronic conditions (61, 62). The occurrence of specific complications diminished the scores of the SF-36 scale, for example, VOC, asthma, and avascular necrosis (63).

The VOCs are the key determinant factors of patients’ HRQOL. Frequent and severe VOCs impact multiple domains of HRQOL (emotion, social functioning, stiffness, sleep, and pain) and work productivity (64). In both young and adult patients visiting the emergency department for acute pain crises, adequate pain treatment improves HRQOL scores at discharge (62–65).

Chronic pain is a major cause of impaired HRQOL, and high opioid usage appears to have a negative impact on HRQOL: patients prescribed ≥ 90 morphine milligram equivalents have significantly lower SF-36 scores (66).

The presence of pain on 3 or more days a week is associated with worse pain interference and anxiety, with impairment of HRQOL even in young individuals (67, 68). Furthermore, persistent pain has neurocognitive and psychological impacts (69).

The presence of neuropathic pain in adolescents is associated with poor HRQOL (70). Fatigue appears to be another major determinant of lower HRQOL, since fatigue interferes with school, work, and exercise (71). The impact of somatic symptom burden (SSB) on pain, depression, anxiety, healthcare utilization, and HRQOL has been prospectively studied. High somatic symptom burden was 1.5–2 times more prevalent in SCD patients than in primary care, showing that SCD patients tend to be high somatizers (72).

Depression and anxiety are common in SCD and have a negative impact on daily pain and physical and mental HRQOL, with poor functioning in all SF-36 subscales (73). In young SCD patients, the use of maladaptive emotion regulation strategies (e.g., keeping the emotions to oneself) and pain catastrophizing (negative “mental set” during actual or anticipated pain experience) is associated with increased symptoms of anxiety, depression, and poor HRQOL (74, 75). Even in adult cohorts, catastrophizing was correlated with poorer mental and HRQOL (76). Sleep disturbances are a consistent problem among SCD patients. In a cohort of adults with SCD undergoing stem cell transplant, 17% of the participants had sleep disruptions (77).

The relationship between HRQOL and treatments, hydroxyurea (HU) or chronic red cell transfusion (CRCT), has been investigated.

HU improves some aspects of HRQOL in adult patients, with benefits in social function, pain recall. and general health perception (78). Worse HRQOL scores have been reported when adherence is low (79). HU adherence rate is multifactorial: adherence barriers that have been identified for HU are negative beliefs (patients reporting that HU does not work or they do not know if it bothers them), recall barriers (difficulties in remembering to take the dose), and access barriers (reported difficulties in paying for HU or getting refills on time). Young patients with fewer barriers to HU adherence have higher adherence rates and HRQOL scores (80–82). However, young patients with frequent hospitalizations, emergency room visits, and longer length of stay at the hospital have lower adherence to treatment with an impact on multiple HRQOL domains (83).

Children undergoing CRCT therapy have significantly higher self-reported HRQL scores for domains of pain and pain-related functioning, compared with children not undergoing CRCT. Additionally, they appear to have fewer worries about SCD-related complications (84).

#### Europe

We found only one study that was conducted in the European region, and it was about the internal validity and reliability of ASCQ-Me measures for use in the United Kingdom SCD population (59).

### Thalassemia

This review analyzed 36 studies investigating HRQOL in children, adolescents, and adult patients affected by beta-thalassemia worldwide. Supplementary material 3 (Tables 2.1 and 2.2) summarize the characteristics of thalassemia studies that have been selected.

The examination of HRQOL was conducted through the submission of different generic and disease-specific questionnaires.

The SF-36 represents the most used generic tool for HRQOL assessment in thalassemia patients (31/36 studies, 86.1%). The PedsQL was performed in two studies (5.6%). The TranQoL disease-specific measure was used in nine studies (16.7%).

Other questionnaires were used in association with the above-mentioned ones (36.1%): CHQ, SICT, SCL 90, and BDI (eight studies); BAI, BFI, BPI SF, LSI, HUI 3, DAS21, HADS, PSQI, MSPSS, WCQ, and FACT-BMT (one study for each one) (for full names of questionnaires, see Supplementary material 1–Table 1).

Several studies (17/36 studies, 47.2%) adopted questionnaires translated into local languages and previously tested for consistency and reliability: the SF-36 was translated into six languages (Greek, Italian, Persian, Arabic, Turkish, and Malaysian) and the TranQoL was translated in two languages (Persian and Greek).

Most studies were conducted in Middle East countries (21/36 studies, 58.3%) and Europe [9/36 studies (25%), including two studies conducted in Europe, the United States, and Canada], especially in the Mediterranean region; few studies took place in the United States and Canada (4/36 studies, 11.1%). Only three studies were conducted in South-East Asia and none in Africa.

A meta-analysis on HRQOL of patients with beta-thalassemia major was assessed by using SF-36. The results suggested that beta-thalassemia major had a significantly negative effect on physical and mental health status (11).

#### Risk of bias assessment

Our review included 34 observational studies (94.4%), 1 randomized clinical trial (0.2%), and 1 systematic review (0.2%). The overall risk of bias assessment was low (Supplementary material 2–Table 2).

##### Risk of bias assessment for observational studies

A Critical Appraisal checklist for cross-sectional and prospective observational studies and related risk of bias assessment results are available in Supplementary material 2 (Figures 1, 2 and Table 2). Among 32 cross-sectional observational studies (32/34, 94%), 28 were rated “good,” and 4 were rated “fair.” Prospective observational studies were two (2/34, 6%), of which one was rated “good” and one was rated “fair.”

##### Risk of bias assessment for clinical trials

The risk of bias assessment for the only randomized clinical trial is available in Supplementary material 2 (Figure 3 and Table 2) and was rated “good.”

##### Risk of bias assessment for systematic review

The risk of bias assessment for the only systematic review is available in Supplementary material 2 (Figure 5 and Table 2) and was rated “good.”

#### Middle East

Most studies were conducted in Middle East countries (21/36 studies): 12 studies took place in Iran, 3 in Pakistan, 3 in Turkey, 2 in Saudi Arabia, and 1 in Lebanon.

The selected studies involved different age groups. Mixed populations, including children, adolescents, and adults, were investigated in 19/21 studies (90,5%), while 2 studies involved only adults (9.5%).

The HRQOL assessment was performed through different tools: 17/21 studies (81%) administered the SF-36 questionnaire, 4/21 studies (19%) applied the TransQoL, and 6/21 studies (28,6%) added other instruments, such as DAS21, PSQI, BDI, SICT, HADS, BAI, SCL-90-R, LSI, and MSPSS.

The Persian version of the SF-36 questionnaire is a reliable and valid instrument (85, 86). However, a new questionnaire with five subscales and 20 questions was structured to better analyze the patient outcomes (87).

The HRQOL assessment in patients with thalassemia major was better than those with thalassemia intermedia with regard to physical functioning, emotional, and mental health domains (88, 89).

Several studies investigated HRQOL domain determining factors in thalassemia. Physical functioning was significantly compromised in older patients and in those reporting chronic pain and a history of splenectomy (90, 91).

Disease complications, poor compliance with iron-chelating therapy, and poor economic status were associated with HRQOL impairment (85–90). Decreased smell function emerged as an additional complication of this disease, leading to poor HRQOL (92).

Interestingly, receiving hematopoietic stem cell transplantation (HSCT) and living in rural areas are factors related to improved HRQOL (91). In addition to clinical conditions, it appears that alterations in lifestyle, social relationships, and psychological events showed improvement in treatment outcomes (93, 94). Furthermore, it was observed that regular exercise programs combined with drug therapy and blood transfusion can improve HRQOL in beta-thalassemia patients (95).

An Iranian study examined concerns about infections occurring due to regular blood transfusion and the use of blood products, which was assessed through the submission of TranQoL (96). Transfusion-dependent infections led to a poor HRQOL (97).

The majority of beta-thalassemia major patients suffered from mild to severe depression, anxiety, and stress. These conditions have been confirmed to affect several life functions (98–101) and to predispose the development of psychiatric symptoms with the need for appropriate psychiatric counseling (102). Specific instruments, such as DAS 21, BAI, and BDI, have been used to better examine these conditions (98, 99, 101, 103). Mental and physical quality of life scores were predicted by symptoms of depression and somatic comorbidities, while total sleep quality was predicted by anxiety symptoms and somatic comorbidities (101, 103).

A cross-sectional study involving adolescent and adult patients suffering from beta-thalassemia major was conducted in Turkey with the administration of the Beck Depression Inventory (BDI) and SF-36. Social economic status, being married and single, and comorbidities emerged to impact differently on HRQOL; mental health and social functioning scores were better in working patients, whereas emotional role scores were worse, and comorbidities influenced physical functioning and bodily pain scores (93).

In Saudi Arabia, a discrepancy in HRQOL-specific domains has been shown in different regions: social and emotional functioning scores were lower in non-Saudis, particularly in females, whereas physical domain was more compromised in Saudis (104).

#### United States and Canada

In the United States and Canada, four studies have been conducted. The SF-36 has been adopted in 3/4 of studies (75%), the PedsQL in 1/4 study (25%), and the TransQoL in 2/4 of studies (50%).

The selected studies involved different age groups: mixed populations including children, adolescents, and adults were investigated in 3/4 studies (75%), while 1 study involved adolescents and adults (25%).

The TransQol emerged to be a valid and reliable tool, showing a substantial agreement with the HUI3, the SF-36, and the PedsQL. Moreover, the TranQoL can measure responsiveness to transfusion therapy in time, with some evidence of superior characteristics when compared to generic tools, such as HUI3 and PedsQL (28).

Innovative methods of survey, such as smartphone applications, were applied to assess patient and caregiver reported burden of transfusion-dependent beta-thalassemia in the United States, Canada, and Italy. A prospective and observational study showed that the transfusion dependency burden was driven by high time spent on disease management, fatigue, and pain symptoms (105).

Physical and general health domains were mostly impaired in another study, while the scores associated with emotional and social domains do not stand out as significantly compromised (106, 107).

#### Europe

Different studies were conducted in Europe [9/36 studies (25%)] in order to investigate HRQOL and associated factors: four studies were conducted in Greece, three in Italy, one in Italy, the United Kingdom, and the United States, and one in the United Kingdom, the United States, and Canada.

The SF-36 was employed in 8/9 studies (89%), the TranQoL in 1 study, and 4/9 studies (44.4%) submitted additional tools (FACT-BMT, SICT, SCL 90, WCQ, BFI, and BPI-SF).

Several studies aimed to evaluate the impact of novel therapeutic strategies on HRQOL. The introduction of deferasirox showed an improvement, particularly in the Mental Health Scale (108, 109). The PRO substudy within the EPIC trial included participating study sites in Australia, Belgium, France, Germany, Greece, Italy, Netherlands, and the United Kingdom. It was conducted to assess self-reported HRQOL and treatment satisfaction, adherence, and persistence in patients with transfusion-dependent iron overload. Observations of the EPIC study suggest that deferasirox was associated with directional improvements in all facets of HRQOL, as assessed by the SF-36 (110).

Moreover, data collected more than 20 years after HSCT showed that the long-term HRQoL of ex-thalassemia patients was very similar to that of the general population. Clinical meaningful differences were only found for general health, whereas the development of GVHD and older age at transplantation were important impairing factors (111). According to this evidence, non-transplanted thalassemia patients showed clinically meaningful differences in physical functioning, body pain, and emotional functioning (112).

In Greece, the stage of heart failure in adult patients emerged as one of the most important factors that are negatively related to physical functioning, role-physical, general health, social functioning, and vitality (12).

In Italy, close attention was paid to psychopathological features of adult patients who underwent regular blood transfusions. The administration of targeted instruments, such as SCL90, in addition to SF-36 demonstrated the prevalence of somatization, obsessive-compulsive disorder, and depression. These findings were associated with the evidence of limitations due to emotional problems and social functioning in SF-36 (113).

#### South-East Asia

A literature review of pediatric journals revealed a relative lack of studies that address HRQOL among children with thalassemia in South-East Asia. Three studies summarize patients’ perceptions of daily functioning and were conducted in Singapore, India, and Malaysia.

Different questionnaires were submitted to children and caregivers: SF-36 (66.7%), TransQoL, PedsQL, and MOS SF-36.

In Singapore, increasing age and reception of social assistance were the only factors that were significantly associated with lower overall TranQoL scores. Endocrine complications and older age were associated with lower TranQoL scores; however, they did not show statistical significance (114).

A cross-sectional study was conducted in India through the administration of the PedsQL and the SF-36 to children and their caregivers. Data underline low scores in physical, emotional, school, and psychosocial functioning domains in both child-self reports and parent-proxy reports. In child reports, school functioning was affected by their condition, while there was no significant difference in scores in the domain of social functioning (115).

In Malaysia, a significant proportion of transfusion-dependent thalassemia patients are not prescribed desferrioxamine, due to its high cost. By having higher income, thalassemia patients not only would use desferrioxamine at the optimum dose, but they would also initiate desferrioxamine injections earlier, as observed in this study. This emerged to be associated with higher HRQOL (116).

## Discussion

The WHO has defined hemoglobinopathies as public health problems at a global level (117), due to population movements (118), consequent change in epidemiology, and increased life expectancy (2). Both SCD and thalassemia are chronic diseases with potential acute and chronic complications that impair HRQOL. Thus, the follow-up of patients affected by hemoglobinopathies should be holistic and comprehensive (117). Several Global Health Organizations and patient groups are trying to assess gaps in treatment and care and plan specific interventions at a global level (117). The aim of our review was to evaluate if and how HRQOL is assessed at the global level. Specific comments for SCD and thalassemia are discussed separately.

### Sickle cell disease

The HRQOL of SCD patients has been assessed in all continents where the disease is prevalent. However, there is a huge disparity in the methodology and the quantity of HRQOL studies in different countries.

The most adopted questionnaires in the studies included in our search are SF-36 (39%), PROMIS (29%), and PedsQL (23%), which are generic questionnaires for HRQOL assessment. Disease-specific questionnaires, such as ASCQ-Me and SCSES, are less used (12 and 3%, respectively), probably because they have been recently developed and have not been validated for use in many clinical research settings (27) or are less known by clinicians (3). Study populations mainly comprised adults (41%) or children and adolescents (32%). Few studies included only children with age ≤ 14 years (6%).

Only four studies on HRQOL were conducted in the Middle East and four studies in Africa, although SCD is a frequent disease in these areas (1). This highlights the need to reinforce HRQOL evaluation in these settings.

Despite SCD being one of the most frequent monogenic diseases in Europe (1), we found only one paper on HRQOL, which was conducted in the United Kingdom and focused only on the validity of ASCQ-Me measures (59). A possible reason for such scarcity could be that in hospital-centered health systems like the European systems, clinicians’ choices are more guided by clinical and hematological parameters rather than by systematic HRQOL evaluation (3, 119). Moreover, many European patients with SCD are immigrants from low-income countries. Hence, clinicians may assume that their HRQOL will surely improve after the onset of treatment in Europe (10, 118, 120). In addition, it may be easier to evaluate standardized clinical and hematological outcomes compared to the ones related to HRQOL, due to linguistic, social, or cultural barriers (3).

Interestingly, different outcomes have been found in different settings. In Middle East countries like Oman and Iran where rapid access to cure is not always feasible, attention is being given to educate patients and caregivers regarding pain treatment at home and principles to prevent complications. A self-management and educational program designed for the needs of patients can be effective in motivating them to change their behavior and thus promote the HRQOL (32, 33).

We found that HRQOL in African cohorts is low, and there is a high prevalence of chronic complications (49). Moreover, it seems that Mozambican SCD children and their caregivers have lower HRQOL in emotional and communication domains than in pain-related domains (50). A study on factors influencing hopelessness has been conducted in Nigeria, which showed that the main driving factors are hemoglobin concentration, resilience, and depression (51). Such aspects suggest the need for psychological support in these regions.

Even in Brazil, HRQOL of SCD patients and caregivers is low and is influenced by psychological aspects, such as having perceived any kind of prejudice in life. Psychological factors are a central problem in SCD patients, and there is a need for health promotion strategies aimed specifically at the amelioration of psychological variables (48).

In low-income areas. such as in the Brazilian regions, the HRQOL of patients varies according to the health center they visit for treatment, suggesting a huge disparity of treatments between rich and poor areas. The presence of rural–urban differences in low-income countries like Jamaica can have an impact on the HRQOL of patients; patients in urban areas have worse HRQOL scores. A reason could be the lack of family and social support for patients who have moved from rural to urban areas (42).

In the United States, in contrast to other countries, many HRQOL studies are focused on the relationship between disease-related complications and HRQOL, with VOC, asthma, and avascular necrosis being the main impacting complications. Many studies focus on HRQOL and treatment adherence or the amount of drugs used. The HU adherence level is multifactorial (80–82), and it has been demonstrated that a good adherence improves HRQOL with benefits in social function, pain recall, and general health perception (78). The need for opioid usage in patients with chronic pain diminishes HRQOL (66), with an impact also on depression, anxiety, neuropsychological abilities, and sleep (70, 73, 77). Since somatic symptom burden is highly prevalent in SCD patients (72), patients may benefit from treatments shown to be beneficial in the treatment of somatoform disorders (e.g., psychotherapy and medications). Optimal management of pain and opioid usage and good HU adherence may help decrease the overall burden on patients and healthcare systems.

### Beta-thalassemia

Beta-thalassemia is a disorder related to hemoglobin, which is prevalent in the communities of the Indian subcontinent, the Mediterranean region, the Middle East, and South-East Asia. Moreover, this disease shows a significant incidence in some parts of Africa (121). This review collected several studies related to HRQOL. Our findings partially mirror the geographic prevalence of beta-thalassemia: most studies of HRQOL were conducted in Middle East countries and in Southern Europe, and a few studies took place in the United States and Canada.

In contrast with the beta-thalassemia epidemiology and related disease burden, HRQOL emerged to be poorly investigated in South-East Asia and no studies have been found in Africa. This lack of studies addressing HRQOL should be better investigated, in order to improve patient outcomes and to understand eventual critical points in the rehabilitation of thalassemia patients. Social and economic issues faced by the populations of developing countries, including patients and caregivers, could play an important role in affecting access to the sanitary structure, treatment adherence, clinical follow-up, and related lack of data collection (115).

According to thalassemia epidemiology, our data showed predominant attention to explore HRQOL and associated factors in Middle East countries. Several studies aimed to define factors related to specific dimensions of HRQOL as clinical features, ranging from symptoms to choice of therapy and compliance, social, and psychological functioning.

Alongside, the extent of European HRQOL considerations was restricted compared to Middle East countries and mostly limited to Italy and Greece. In addition, the rise in global population movements leads to the spread of the disease to northern and western Europe with consequent implications for European public health (120). This finding suggests that focusing on HRQOL in beta-thalassemia patients represents a big challenge for European healthcare services overall. It should be addressed from a European perspective rather than a merely national one (117). A joint effort between clinical care providers and patient organizations should be enhanced.

Our review showed that the examination of HRQOL has been widely explored through the administration of the SF-36, used as a general research tool to assess health status, and the less represented PedsQL. The TranQoL was used as a disease-specific HRQOL measure for transfusion-dependent thalassemia major patients in several studies, suggesting the need for targeted instruments in HRQOL investigation.

The assessment of HRQOL through generic and specific tools is not widely shared. This review concurrently shows that ancillary questionnaires have been applied in order to properly study specific life domains and related affections in HRQOL. An American study pointed out that TranQoL has some evidence of superior characteristics compared to generic tools like PedsQL (28); whereas an Iranian study tailored a new questionnaire and proved it to be reliable and valid with the aim to better analyze patient outcomes (87).

Thus, the combination between disease-specific questionnaires and well-established generic tools can provide a clear picture of the patient’s perspective.

In the Middle East countries, older patients and associated disease complications, chronic pain, history of splenectomy, poor compliance with iron-chelating therapy, and poor economic status emerged to be related to the impairment of quality of life (85, 90, 91).

In Europe, iron-chelation therapy with deferasirox and HSCT showed to play an important role in HRQOL improvement in multiple areas (108–111).

Transfusion burden and fatigue emerged as significant determinants of HRQOL in different continents, in spite of differences in the healthcare system and organization of care (105, 115). This indicates that they are strongly disease-related and specific actions should be undertaken.

In the United States and Canada, studies about HRQOL in patients with transfusion-dependent thalassemia major reported that physical and general health domains were mostly affected, whereas emotional and social domains were not significantly compromised (106, 107).

In addition to clinical conditions, a great emphasis on psychological features and psychiatric comorbidity was laid in the Middle East and Italy, where the mental and physical quality of life domains were predicted by symptoms of depression and anxiety (98–101, 103) with evidence of limitations due to emotional problems and social functioning (113).

Finally, economic status is an important variable related to HRQOL impairment in developing countries, where psychiatric disorders are widely recognized as disturbing factors in physical and mental domains. Furthermore, regular blood transfusion leads to a high risk of transfusion-mediated infection in thalassemia patients and related HRQOL impairment in developing countries, in addition to common complications, such as iron overload, due to inadequate awareness and poor screening practices (13).

In conclusion, HRQOL is a complex outcome that reflects how chronic disease is experienced by patients. Although it has been increasingly incorporated in clinical research and clinical practice worldwide, it is still not sufficiently evaluated in SCD and thalassemia. General HRQOL questionnaires are not enough to explore disease burden in hemoglobinopathies, and the use of disease-specific ones needs to be stimulated, with translation into local languages if necessary. Further studies are needed to widely explore the proper assessment of HRQOL and to increase our knowledge of HRQOL worldwide.

## Limitations

The main limitation of this review relies on the heterogeneity of the included studies, even if the bias assessment rating is good. In addition, HRQOL assessment is not standardized all over the world, and we found a huge heterogeneity in its evaluation. For this reason, statistical analyses were not conducted.

## Data availability statement

The original contributions presented in this study are included in the article/Supplementary material, further inquiries can be directed to the corresponding authors.

## Author contributions

FR and GB organized the database, wrote first draft of the manuscript, and equally contributed to the review writing. FR wrote sickle cell disease sections. GB wrote thalassemia sections. RC supervised the work. All authors contributed to conception and design of the work and manuscript revision, read, and approved the submitted version.
