# Supplementary material for: Health-related quality of life in hemoglobinopathies: A systematic review from a global perspective
Source: Front Pediatr. 2022 Aug 25;10:886674. doi: 10.3389/fped.2022.886674 (PMC9452907; doi:10.3389/fped.2022.886674)
Supplement: Supplementary file 1 [file Data_Sheet_1.PDF]

# Supplementary materials 1

## Fig. 1 - SF-36 questionnaire.

### SF-36 QUESTIONNAIRE

**Name:**____________________ **Ref. Dr:**___________________ **Date:** _______

**ID#:** _______________ **Age:** _______ **Gender: M / F**

Please answer the 36 questions of the **Health Survey** completely, honestly, and without interruptions.

**GENERAL HEALTH:**
**In general, would you say your health is:**
◯ Excellent ◯ Very Good ◯ Good ◯ Fair ◯ Poor

**Compared to one year ago, how would you rate your health in general now?**
◯ Much better now than one year ago
◯ Somewhat better now than one year ago
◯ About the same
◯ Somewhat worse now than one year ago
◯ Much worse than one year ago

**LIMITATIONS OF ACTIVITIES:**
The following items are about activities you might do during a typical day. Does your health now limit you in these activities? If so, how much?

**Vigorous activities, such as running, lifting heavy objects, participating in strenuous sports.**
◯ Yes, Limited a lot ◯ Yes, Limited a Little ◯ No, Not Limited at all

**Moderate activities, such as moving a table, pushing a vacuum cleaner, bowling, or playing golf**
◯ Yes, Limited a Lot ◯ Yes, Limited a Little ◯ No, Not Limited at all

**Lifting or carrying groceries**
◯ Yes, Limited a Lot ◯ Yes, Limited a Little ◯ No, Not Limited at all

**Climbing several flights of stairs**
◯ Yes, Limited a Lot ◯ Yes, Limited a Little ◯ No, Not Limited at all

**Climbing one flight of stairs**
◯ Yes, Limited a Lot ◯ Yes, Limited a Little ◯ No, Not Limited at all

**Bending, kneeling, or stooping**
◯ Yes, Limited a Lot ◯ Yes, Limited a Little ◯ No, Not Limited at all

**Walking more than a mile**
◯ Yes, Limited a Lot ◯ Yes, Limited a Little ◯ No, Not Limited at all

**Walking several blocks**
◯ Yes, Limited a Lot ◯ Yes, Limited a Little ◯ No, Not Limited at all

**Walking one block**
◯ Yes, Limited a Lot ◯ Yes, Limited a Little ◯ No, Not Limited at all

**Bathing or dressing yourself**
◯Yes, Limited a Lot ◯Yes, Limited a Little ◯No, Not Limited at all

**PHYSICAL HEALTH PROBLEMS:**
During the past 4 weeks, have you had any of the following problems with your work or other regular daily activities as a result of your physical health?

**Cut down the amount of time you spent on work or other activities**
◯Yes ◯No

**Accomplished less than you would like**
◯Yes ◯No

**Were limited in the kind of work or other activities**
◯Yes ◯No

**Had difficulty performing the work or other activities (for example, it took extra effort)**
◯Yes ◯No

**EMOTIONAL HEALTH PROBLEMS:**
During the past 4 weeks, have you had any of the following problems with your work or other regular daily activities as a result of any emotional problems (such as feeling depressed or anxious)?

**Cut down the amount of time you spent on work or other activities**
◯Yes ◯No

**Accomplished less than you would like**
◯Yes ◯No

**Didn't do work or other activities as carefully as usual**
◯Yes ◯No

**SOCIAL ACTIVITIES:**
**Emotional problems interfered with your normal social activities with family, friends, neighbors, or groups?**

◯Not at all ◯Slightly ◯Moderately ◯Severe ◯Very Severe

**PAIN:**
**How much bodily pain have you had during the past 4 weeks?**

◯None ◯Very Mild ◯Mild ◯Moderate ◯Severe ◯Very Severe

**During the past 4 weeks, how much did pain interfere with your normal work (including both work outside the home and housework)?**

◯Not at all ◯A little bit ◯Moderately ◯Quite a bit ◯Extremely

**ENERGY AND EMOTIONS:**

These questions are about how you feel and how things have been with you during the last 4 weeks. For each question, please give the answer that comes closest to the way you have been feeling.

**Did you feel full of pep?**

- All of the time
- Most of the time
- A good Bit of the Time
- Some of the time
- A little bit of the time
- None of the Time

**Have you been a very nervous person?**

- All of the time
- Most of the time
- A good Bit of the Time
- Some of the time
- A little bit of the time
- None of the Time

**Have you felt so down in the dumps that nothing could cheer you up?**

- All of the time
- Most of the time
- A good Bit of the Time
- Some of the time
- A little bit of the time
- None of the Time

**Have you felt calm and peaceful?**

- All of the time
- Most of the time
- A good Bit of the Time
- Some of the time
- A little bit of the time
- None of the Time

**Did you have a lot of energy?**

- All of the time
- Most of the time
- A good Bit of the Time
- Some of the time
- A little bit of the time
- None of the Time

**Have you felt downhearted and blue?**

- All of the time
- Most of the time
- A good Bit of the Time
- Some of the time
- A little bit of the time
- None of the Time

**Did you feel worn out?**

- All of the time
- Most of the time
- A good Bit of the Time
- Some of the time
- A little bit of the time
- None of the Time

**Have you been a happy person?**

- All of the time
- Most of the time
- A good Bit of the Time
- Some of the time
- A little bit of the time
- None of the Time

**Did you feel tired?**

- All of the time
- Most of the time
- A good Bit of the Time
- Some of the time
- A little bit of the time
- None of the Time

**SOCIAL ACTIVITIES:**
**During the past 4 weeks, how much of the time has your physical health or emotional problems interfered with your social activities (like visiting with friends, relatives, etc.)?**

- All of the time
- Most of the time
- Some of the time
- A little bit of the time
- None of the Time

**GENERAL HEALTH:**
**How true or false is each of the following statements for you?**

**I seem to get sick a little easier than other people**
Definitely true | Mostly true | Don't know | Mostly false | Definitely false

**I am as healthy as anybody I know**
Definitely true | Mostly true | Don't know | Mostly false | Definitely false

**I expect my health to get worse**
Definitely true | Mostly true | Don't know | Mostly false | Definitely false

**My health is excellent**
Definitely true | Mostly true | Don't know | Mostly false | Definitely false

**Fig.2 - PROMIS questionnaire (short form).**

| | Please respond to each item by marking <u>one box per row</u> | Excellent | Very good | Good | Fair | Poor |
|---|---|---|---|---|---|---|
| Global 01 | In general, would you say your health is: | ☐ 5 | ☐ 4 | ☐ 3 | ☐ 2 | ☐ 1 |
| Global 02 | In general, would you say your quality of life is: | ☐ 5 | ☐ 4 | ☐ 3 | ☐ 2 | ☐ 1 |
| Global 03 | In general, how would you rate your physical health? | ☐ 5 | ☐ 4 | ☐ 3 | ☐ 2 | ☐ 1 |
| Global 04 | In general, how would you rate your mental health, including your mood and your ability to think? | ☐ 5 | ☐ 4 | ☐ 3 | ☐ 2 | ☐ 1 |
| Global 05 | In general, how would you rate your satisfaction with your social activities and relationships? | ☐ 5 | ☐ 4 | ☐ 3 | ☐ 2 | ☐ 1 |
| Global 09 | In general, please rate how well you carry out your usual social activities and roles. (This includes activities at home, at work and in your community, and responsibilities as a parent, child, spouse, employee, friend, etc.) | ☐ 5 | ☐ 4 | ☐ 3 | ☐ 2 | ☐ 1 |
| | | | | | | |
| | | Completely | Mostly | Moderately | A Little | Not At All |
| Global 06 | To what extent are you able to carry out your everyday physical activities such as walking, climbing stairs, carrying groceries, or moving a chair? | ☐ 5 | ☐ 4 | ☐ 3 | ☐ 2 | ☐ 1 |
| | | | | | | |
| | **In the past 7 days** | Never | Rarely | Sometimes | Often | Always |
| Global 10 | How often have you been bothered by emotional problems such as feeling anxious, depressed or irritable? | ☐ 5 | ☐ 4 | ☐ 3 | ☐ 2 | ☐ 1 |
| | | None | Mild | Moderate | Severe | Very Severe |
| Global 08 | How would you rate your fatigue on average? | ☐ 5 | ☐ 4 | ☐ 3 | ☐ 2 | ☐ 1 |
| Global 07 | How would you rate your pain on average? | ☐ 0 ☐ 1 ☐ 2 ☐ 3 ☐ 4 ☐ 5 ☐ 6 ☐ 7 ☐ 8 ☐ 9 ☐ 10<br>No Pain … Worst Imaginable Pain | | | | |

***Scoring:***

Re-code Global07. The recoded score ranges from 1 to 5.
(0 No pain =**5**; 1, 2, or 3 =**4**; 4, 5, or 6 =**3**; 7, 8, or 9 =**2**; 10 worst pain imaginable =**1**)

After recoding, the
Global Physical Health score = SUM responses to G03 + G06 + G07 + G08.
Global Mental Health score = SUM G02 + G04 + G05 + Global10.

**Fig. 3 - PedsQL questionnaire.**

| Exemplary Item Description (0–4 points)* |
|---|
| physical functioning |
| 1. It is hard for me to walk more than one block |
| 2. It is hard for me to run |
| 3. It is hard for me to do sports activity or exercise |
| 4. It is hard for me to lift something heavy |
| 5. It is hard for me to take a bath or shower by myself |
| 6. It is hard for me to do chores around the house |
| 7. I hurt or ache |
| 8. I have low energy |
| psychosocial functioning |
| emotional functioning |
| 1. I feel afraid or scared |
| 2. I feel sad or blue |
| 3. I feel angry |
| 4. I have trouble sleeping |
| 5. I worry about what will happen to me |
| social functioning |
| 1. I have trouble getting along w/ others |
| 2. Others do not want to be my friend |
| 3. Others tease me |
| 4. I cannot do things others my age can do |
| 5. It is hard to keep up w/ my peers |
| school functioning |
| 1. It is hard to pay attention at work/school |
| 2. I forget things |
| 3. I have trouble keeping up w/ my work or studies |
| 4. I miss work or school because of not feeling well |
| 5. I miss work or school to go to the doctor or hospital |

* Wording of items is adapted to the age groups of the PedsQL 4.0

**Fig. 4 - SCSES questionnaire.**

| | NOT AT ALL SURE | NOT SURE | NEITHER | SURE | VERY SURE |
|---|---|---|---|---|---|
| A. **HOW SURE ARE YOU** THAT YOU CAN DO SOMETHING TO CUT DOWN ON MOST OF THE PAIN YOU HAVE WHEN HAVING A PAIN EPISODE? | ☐ | ☐ | ☐ | ☐ | ☐ |
| B. **HOW SURE ARE YOU** THAT YOU CAN KEEP DOING MOST OF THE THINGS YOU DO DAY-TO-DAY? | ☐ | ☐ | ☐ | ☐ | ☐ |
| C. **HOW SURE ARE YOU** THAT YOU CAN KEEP SICKLE CELL DISEASE PAIN FROM INTERFERING WITH YOUR SLEEP? | ☐ | ☐ | ☐ | ☐ | ☐ |
| D. **HOW SURE ARE YOU** THAT YOU CAN REDUCE YOUR SICKLE CELL DISEASE PAIN BY USING METHODS OTHER THAN TAKING EXTRA MEDICATION? | ☐ | ☐ | ☐ | ☐ | ☐ |
| E. **HOW SURE ARE YOU** THAT YOU CAN CONTROL HOW OFTEN OR WHEN YOU GET TIRED? | ☐ | ☐ | ☐ | ☐ | ☐ |
| F. **HOW SURE ARE YOU** THAT YOU CAN DO SOMETHING TO HELP YOURSELF FEEL BETTER IF YOUR ARE FEELING SAD OR BLUE? | ☐ | ☐ | ☐ | ☐ | ☐ |
| G. AS COMPARED WITH OTHER PEOPLE WITH SICKLE CELL DISEASE, **HOW SURE ARE YOU** THAT YOU CAN MANAGE YOUR LIFE FROM DAY-TO-DAY? | ☐ | ☐ | ☐ | ☐ | ☐ |
| H. **HOW SURE ARE YOU** THAT YOU CAN MANAGE YOUR SICKLE CELL DISEASE SYMPTOMS SO THAT YOU CAN DO THE THINGS YOU ENJOY DOING? | ☐ | ☐ | ☐ | ☐ | ☐ |
| I. **HOW SURE ARE YOU** THAT YOU CAN DEAL WITH THE FRUSTRATION OF HAVING SICKLE CELL DISEASE? | ☐ | ☐ | ☐ | ☐ | ☐ |

**Fig. 5 - ASCQ-Me questionnaire.**

ASCQ-Me Emotional Impact

**Survey Instructions**

- Answer all the questions by checking the box to the left of your answer.

**1. In the past 7 days, how often did you feel completely hopeless because of your health?**

- ☐ Never
- ☐ Rarely
- ☐ Sometimes
- ☐ Often
- ☐ Always

**2. In the past 7 days, how lonely did you feel because of your health problems?**

- ☐ Not at all
- ☐ A little
- ☐ Somewhat
- ☐ Quite
- ☐ Very

**3. In the past 7 days, how depressed were you about your health problems?**

- ☐ Not at all
- ☐ A little
- ☐ Somewhat
- ☐ Quite
- ☐ Very

**4. In the past 7 days, how much did you worry about getting sick?**

- ☐ Not at all
- ☐ A little bit
- ☐ Somewhat
- ☐ Quite a bit
- ☐ Very much

**5. In the past 7 days, how often were you very worried about needing to go to the hospital?**

- ☐ Never
- ☐ Rarely
- ☐ Sometimes
- ☐ Often
- ☐ Always

©American Institutes for Research Version 2.0

# ASCQ-Me Pain Impact

**Survey Instructions**

- Answer all the questions by checking the box to the left of your answer.

**1. In the past 7 days, how often did you have pain so bad that you could not do anything for a whole day?**

- ☐ Never
- ☐ Rarely
- ☐ Sometimes
- ☐ Often
- ☐ Always

**2. In the past 7 days, how often did you have pain so bad that you could not get out of bed?**

- ☐ Never
- ☐ Rarely
- ☐ Sometimes
- ☐ Often
- ☐ Always

**3. In the past 7 days, how often did you have very severe pain?**

- ☐ Never
- ☐ Rarely
- ☐ Sometimes
- ☐ Often
- ☐ Always

**4. In the past 7 days, how often did you have pain so bad that you had to stop what you were doing?**

- ☐ Never
- ☐ Rarely
- ☐ Sometimes
- ☐ Often
- ☐ Always

**5. In the past 7 days, how often did you have pain so bad that it was hard to finish what you were doing?**

- ☐ Never
- ☐ Rarely
- ☐ Sometimes
- ☐ Often
- ☐ Always

## ASCQ-Me SCD Medical History Checklist

**1. Have you ever had open sores on your legs or feet (leg ulcers)?**

☐ Yes

☐ No

**2. Has a doctor or nurse ever told you that you have lung damage?**

☐ Yes

☐ No

**3. Has a doctor or nurse ever told you that you have kidney damage?**

☐ Yes

☐ No

**4. Has a doctor or nurse ever told you that you have eye damage called retinopathy?**

☐ Yes

☐ No

**5. Has a doctor or nurse ever told you that you have damage to your hip or shoulder due to sickle cell disease?**

☐ Yes

☐ No

**6. Has a doctor or nurse ever told you that you have had a stroke?**

☐ Yes

☐ No

**7. Has your spleen either been removed or seriously damaged due to sickle cell disease?**

☐ Yes

☐ No

**8. Do you get regular blood transfusions for your sickle cell disease?**

☐ Yes

☐ No

**9. Do you take pain medicine every day for your sickle cell disease?**

☐ Yes

☐ No

**Thank you!**

© 2010-2020 American Institutes for Research

## ASCQ-Me Sleep Impact

**Survey Instructions**

- Answer all the questions by checking the box to the left of your answer.

**1. In the past 7 days, how often did you stay up most of the night because you could not fall asleep?**

☐ Never
☐ Rarely
☐ Sometimes
☐ Often
☐ Always

**2. In the past 7 days, how often was it very easy for you to fall asleep?**

☐ Never
☐ Rarely
☐ Sometimes
☐ Often
☐ Always

**3. In the past 7 days, how often did you have a lot of trouble falling asleep?**

☐ Never
☐ Rarely
☐ Sometimes
☐ Often
☐ Always

**4. In the past 7 days, how often did you stay up all night because you could not fall asleep?**

☐ Never
☐ Rarely
☐ Sometimes
☐ Often
☐ Always

**5. In the past 7 days, how often did you stay up half of the night because you could not fall asleep?**

☐ Never
☐ Rarely
☐ Sometimes
☐ Often
☐ Always

©American Institutes for Research

# ASCQ-Me Social Functioning Impact

**Survey Instructions**

- Answer all the questions by checking the box to the left of your answer.

**1. In the past 30 days, how much did you rely on others to take care of you because of your health?**

- ☐ Not at all
- ☐ A little bit
- ☐ Somewhat
- ☐ Quite a bit
- ☐ Very much

**2. In the past 30 days, how often did your health slow you down?**

- ☐ Never
- ☐ Rarely
- ☐ Sometimes
- ☐ Often
- ☐ Always

**3. In the past 30 days, how often did your health make it hard for you to do things?**

- ☐ Never
- ☐ Rarely
- ☐ Sometimes
- ☐ Often
- ☐ Always

**4. In the past 30 days, how often did your health keep you from going out?**

- ☐ Never
- ☐ Rarely
- ☐ Sometimes
- ☐ Often
- ☐ Always

**5. In the past 30 days, how much did your health make it hard for you to do things with your friends?**

- ☐ Not at all
- ☐ A little bit
- ☐ Somewhat
- ☐ Quite a bit
- ☐ Very much

©American Institutes for Research

**Table 1 - Legend of disease specific questionnaire in beta thalassemia.**

| |
|---|
| Child Health Questionnaire (CHQ) |
| Satisfaction Iron Chelation Therapy (SICT) |
| Symptoms Checklist 90 (SCL 90) |
| Beck Depression Inventory (BDI) |
| Beck Anxiety Inventory (BAI) |
| Beck Five Inventory (BFI) |
| Brief Pain Inventory Short Form (BPI SF) |
| Life Style Inventory (LSI) |
| Health Utilities Index (HUI 3) |
| Depression Anxiety and Stress Scale (DAS21) |
| Hospital Anxiety and Depression Scale (HADS) |
| Pittsburgh Sleep Quality Index (PSQI) |
| Multidimensional Scale of Perceived Social Support (MSPSS) |
| Ways of Coping Questionnaire (WCQ) |
| Functional Assessment of Cancer Transplantation - Bone Marrow Transplantation (FACT-BMT) |
