# Supplementary material for: Health-related quality of life in hemoglobinopathies: A systematic review from a global perspective
Source: Front Pediatr. 2022 Aug 25;10:886674. doi: 10.3389/fped.2022.886674 (PMC9452907; doi:10.3389/fped.2022.886674)
Supplement: Supplementary file 2 [file Data_Sheet_2.PDF]

Supplementary materials 2

Figure 1: JBI - Risk of bias assessment for cross sectional observational studies

	Yes	No	Unclear	Not applicable
1. Were the criteria for inclusion in the sample clearly defined?	☐	☐	☐	☐
2. Were the study subjects and the setting described in detail?	☐	☐	☐	☐
3. Was the exposure measured in a valid and reliable way?	☐	☐	☐	☐
4. Were objective, standard criteria used for measurement of the condition?	☐	☐	☐	☐
5. Were confounding factors identified?	☐	☐	☐	☐
6. Were strategies to deal with confounding factors stated?	☐	☐	☐	☐
7. Were the outcomes measured in a valid and reliable way?	☐	☐	☐	☐
8. Was appropriate statistical analysis used?	☐	☐	☐	☐

Figure 2: JBI - Risk of bias assessment for prospective observational studies

	Yes	No	Unclear	Not applicable
1. Were the two groups similar and recruited from the same population?	☐	☐	☐	☐
2. Were the exposures measured similarly to assign people to both exposed and unexposed groups?	☐	☐	☐	☐
3. Was the exposure measured in a valid and reliable way?	☐	☐	☐	☐
4. Were confounding factors identified?	☐	☐	☐	☐
5. Were strategies to deal with confounding factors stated?	☐	☐	☐	☐
6. Were the groups/participants free of the outcome at the start of the study (or at the moment of exposure)?	☐	☐	☐	☐
7. Were the outcomes measured in a valid and reliable way?	☐	☐	☐	☐
8. Was the follow up time reported and sufficient to be long enough for outcomes to occur?	☐	☐	☐	☐
9. Was follow up complete, and if not, were the reasons to loss to follow up described and explored?	☐	☐	☐	☐
10. Were strategies to address incomplete follow up utilized?	☐	☐	☐	☐
11. Was appropriate statistical analysis used?	☐	☐	☐	☐

s

Figure 3: JBI - Risk of bias assessment for clinical trials

	Yes	No	Unclear	NA
1. Was true randomization used for assignment of participants to treatment groups?	☐	☐	☐	☐
2. Was allocation to treatment groups concealed?	☐	☐	☐	☐
3. Were treatment groups similar at the baseline?	☐	☐	☐	☐
4. Were participants blind to treatment assignment?	☐	☐	☐	☐
5. Were those delivering treatment blind to treatment assignment?	☐	☐	☐	☐
6. Were outcomes assessors blind to treatment assignment?	☐	☐	☐	☐
7. Were treatment groups treated identically other than the intervention of interest?	☐	☐	☐	☐
8. Was follow up complete and if not, were differences between groups in terms of their follow up adequately described and analyzed?	☐	☐	☐	☐
9. Were participants analyzed in the groups to which they were randomized?	☐	☐	☐	☐
10. Were outcomes measured in the same way for treatment groups?	☐	☐	☐	☐
11. Were outcomes measured in a reliable way?	☐	☐	☐	☐
12. Was appropriate statistical analysis used?	☐	☐	☐	☐
13. Was the trial design appropriate, and any deviations from the standard RCT design (individual randomization, parallel groups) accounted for in the conduct and analysis of the trial?	☐	☐	☐	☐

Figure 4: JBI - Risk of bias assessment for quasi experimental studies

	Yes	No	Unclear	Not applicable
1. Is it clear in the study what is the 'cause' and what is the 'effect' (i.e. there is no confusion about which variable comes first)?	☐	☐	☐	☐
2. Were the participants included in any comparisons similar?	☐	☐	☐	☐
3. Were the participants included in any comparisons receiving similar treatment/care, other than the exposure or intervention of interest?	☐	☐	☐	☐
4. Was there a control group?	☐	☐	☐	☐
5. Were there multiple measurements of the outcome both pre and post the intervention/exposure?	☐	☐	☐	☐
6. Was follow up complete and if not, were differences between groups in terms of their follow up adequately described and analyzed?	☐	☐	☐	☐
7. Were the outcomes of participants included in any comparisons measured in the same way?	☐	☐	☐	☐
8. Were outcomes measured in a reliable way?	☐	☐	☐	☐
9. Was appropriate statistical analysis used?	☐	☐	☐	☐

Figure 5: JBI - Risk of bias assessment for systematic reviews

	Yes	No	Unclear	Not applicable
1. Is the review question clearly and explicitly stated?	☐	☐	☐	☐
2. Were the inclusion criteria appropriate for the review question?	☐	☐	☐	☐
3. Was the search strategy appropriate?	☐	☐	☐	☐
4. Were the sources and resources used to search for studies adequate?	☐	☐	☐	☐
5. Were the criteria for appraising studies appropriate?	☐	☐	☐	☐
6. Was critical appraisal conducted by two or more reviewers independently?	☐	☐	☐	☐
7. Were there methods to minimize errors in data extraction?	☐	☐	☐	☐
8. Were the methods used to combine studies appropriate?	☐	☐	☐	☐
9. Was the likelihood of publication bias assessed?	☐	☐	☐	☐
10. Were recommendations for policy and/or practice supported by the reported data?	☐	☐	☐	☐
11. Were the specific directives for new research appropriate?	☐	☐	☐	☐

Figure 6: JBI - Risk of bias assessment for qualitative studies

		Yes	No	Unclear	Not applicable
1.	Is there congruity between the stated philosophical perspective and the research methodology?	☐	☐	☐	☐
2.	Is there congruity between the research methodology and the research question or objectives?	☐	☐	☐	☐
3.	Is there congruity between the research methodology and the methods used to collect data?	☐	☐	☐	☐
4.	Is there congruity between the research methodology and the representation and analysis of data?	☐	☐	☐	☐
5.	Is there congruity between the research methodology and the interpretation of results?	☐	☐	☐	☐
6.	Is there a statement locating the researcher culturally or theoretically?	☐	☐	☐	☐
7.	Is the influence of the researcher on the research, and vice- versa, addressed?	☐	☐	☐	☐
8.	Are participants, and their voices, adequately represented?	☐	☐	☐	☐
9.	Is the research ethical according to current criteria or, for recent studies, and is there evidence of ethical approval by an appropriate body?	☐	☐	☐	☐
10.	Do the conclusions drawn in the research report flow from the analysis, or interpretation, of the data?	☐	☐	☐	☐

Figure 7: JBI - Risk of bias assessment for case report

	Yes	No	Unclear	Not applicable
1. Were patient's demographic characteristics clearly described?	☐	☐	☐	☐
2. Was the patient's history clearly described and presented as a timeline?	☐	☐	☐	☐
3. Was the current clinical condition of the patient on presentation clearly described?	☐	☐	☐	☐
4. Were diagnostic tests or assessment methods and the results clearly described?	☐	☐	☐	☐
5. Was the intervention(s) or treatment procedure(s) clearly described?	☐	☐	☐	☐
6. Was the post-intervention clinical condition clearly described?	☐	☐	☐	☐
7. Were adverse events (harms) or unanticipated events identified and described?	☐	☐	☐	☐
8. Does the case report provide takeaway lessons?	☐	☐	☐	☐

Table 1 - Risk of bias assessment of SCD studies
Legend : y: yes, n: no, NA: not applicable, U: unclear

Author, Country, Year (reference)	Study design	1	2	3	4	5	6	7	8	9	10	11	12	13	Rating
Bulgin D et al, USA, 2019 (7)	cross-sectional pilot study	y	y	y	y	y	y	y	y						good
Singh SA et al, 2020, (15)	narrative review	-	-	-	-	-	-	-	-	-	-	-	-	-	NA, poor
Singh A et al, Wisconsin, 2019, (17)	cross-sectional study	y	y	y	y	n	n	y	y						good
Singh A et al, Wisconsin, 2019, (18)	cross-sectional study	y	y	y	y	y	y	y	y						good
Singh A et al, USA, 2020, (19)	prospective observational study	NA	y	y	y	y	y	y	y	y	y	y			good
Panepinto JA et al, Winsconsin, 2012, (22)	qualitative study	y	y	y	y	y	y	y	y	U	y				good
Panepinto JA et al, USA, 2013, (23)	qualitative study	y	y	y	y	y	U	U	y	y	y				good
Beverung LM et al, USA, 2015 (24)	cross-sectional study	y	y	y	y	y	y	y	y						good
Edwards R et al, USA, 2000 (25)	cross-sectional study	n	y	y	y	n	U	y	y						fair
Keller SD et al, USA, 2014 (26)	cross-sectional study	y	y	y	y	y	y	y	y						good
Treadwell M. J. et al, USA, 2014 (27)	systematic review	y	y	y	y	y	y	U	y	n	y	y			good
Ahmed AE et al, Saudi Arabia, 2015 (30)	cross-sectional study	y	y	y	y	y	y	y	y						good
Khaled A et al, Saudi Arabia, 2021 (31)	cross-sectional study	n	y	y	y	n	U	y	y						fair
Ahmadi M et al, Iran, 2015 (32)	quasi-experimental study	y	y	y	n	y	y	y	y						good
Pandarakutty S et al, Oman, 2019, (33)	quasi-experimental study	y	U	U	y	y	y	y	y						good
Asnani MR et al, Jamaica, 2009 (34)	cross-sectional study	y	y	y	y	y	y	y	y						good
Asnani MR et al, Jamaica, 2007 (35)	cross-sectional study	n	u	y	y	U	U	y	y						fair
Asnani MR, Jamaica, 2009 (36)	cross-sectional study	n	y	y	y	y	y	y	y						good
Menezes AS de O d. P et al, Brazil, 2013 (37)	cross-sectional study	y	n	y	y	y	n	y	y						good
Rodrigues CF de A et al, Brazil, 2021, (38)	cross-sectional study	y	y	y	y	n	n	y	y						good
Oliveira, C. D. L. et al, Brazil, 2019 (39)	cross-sectional study	y	y	y	y	y	y	y	y						good

Osborne JC et al, USA, 2020, (40)	cross-sectional study	n	y	y	y	n	n	y	y						fair
Blake A et al, Jamaica, 2020 (41)	cross-sectional study	U	y	y	y	n	U	y	y						fair
Asnani MR et al, Jamaica, 2008 (42)	cross sectional study	n	y	y	y	y	y	y	y						good
Ohara DG et al, Brazil, 2012 (43)	cross-sectional study	y	n	y	y	n	n	y	y						fair
Goncalves CEA et al, Brazil, 2019 (44)	cross-sectional study	y	y	y	y	y	y	y	y						good
dos Santos JP et al, Brazil, 2013 (45)	cross-sectional study	y	y	U	y	n	U	y	y						fair
Almeida CHS de, Brazil, 2021 (46)	quasi-experimental study	y	y	y	n	y	y	y	y						good
Tinti G et al, Brasil, 2010, (47)	case report	y	n	y	y	y	y	y	y						good
Gibson RC et al, Jamaica, 2013 (48)	cross-sectional study	n	y	y	y	n	n	y	y						fair
Andong AM et al, Cameroon, 2017 (49)	cross-sectional study	y	y	y	y	y	y	y	y						good
Issa F et al, Mozambique, 2020 (50)	cross-sectional study	y	y	y	y	y	y	y	y						good
Aloba O et al, Nigeria, 2020 (51)	cross-sectional study	y	y	y	y	y	y	y	y						good
Ojelabi, AO et al, Nigeria, 2019 (52)	cross-sectional study	y	y	y	y	y	y	y	y						good
Lee S et al, USA, 2020 (53)	systematic review	y	y	y	y	y	y	y	y	n	y	y			good
Knisely MR et al, USA, 2020 (54)	cross-sectional study	y	y	y	y	y	y	y	y						good
Dampier C et al, USA, 2016 (55)	cross-sectional study	y	y	y	y	y	y	y	y						good
Dampier C et al, USA, 2016 (56)	cross-sectional study	n	y	y	y	y	y	y	y						good
Hildenbrand AK et al, USA, 2019 (57)	cross-sectional study	y	y	y	y	y	y	y	y						good
Keller S et al, USA, 2017 (58)	cross-sectional study	y	y	y	y	y	y	y	y						good
Cooper, O. et al, UK, 2019 (59)	cross-sectional study	y	y	y	y	n	U	y	y						good
Curtis S et al, 2017 (60)	systematic review	y	y	y	y	y	U	U	y	n	y	U			poor
McClish DK et al, USA, 2005 (61)	cross-sectional study	y	y	y	y	y	y	y	y						good
Esham KS et al, USA, 2020 (62)	cross-sectional study	y	y	y	y	y	y	y	y						good
Dampier C et al, USA, 2011 (63)	cross-sectional study	n	y	y	y	n	U	y	y						fair

Rizio AA et al, USA, 2020, (64)	cross-sectional study	y	y	y	y	y	y	y	y						good
Brandow AM et al, USA, 2016 (65)	prospective observational study	y	y	y	y	y	y	y	y	y	NA	y			good
Karafin MS et al, USA, 2018 (66)	cross-sectional study	y	y	y	y	y	y	y	y						good
Bakshi N et al, USA, 2018 (67)	cross-sectional study	y	y	y	y	y	y	y	y						good
Badawy SM et al, USA, 2018 (68)	cross-sectional study	y	n	y	y	y	y	n	y						good
Connolly ME et al, USA, 2019 (69)	cross-sectional study	y	y	y	y	y	y	y	y						good
Román ME et al, Winsconsin, 2020, (70)	cross-sectional study	y	y	y	y	n	n	y	y						good
Ameringer S et al, Virginia, 2014, (71)	cross-sectional study	y	y	y	y	n	n	y	y						good
Sogutlu A et al, Virginia, 2011, (72)	cross-sectional study	y	y	y	y	y	y	y	y						good
Levenson, J. L. et al, USA, 2008 (73)	prospective observational study	NA	y	y	y	y	y	y	y	y	y	y			good
Miller M et al, USA, 2021 (74)	cross-sectional study	y	y	y	y	y	y	y	y						good
Bakshi N et al, USA, 2018 (75)	cross-sectional study	y	y	y	y	y	y	y	y						good
Citero V de A et al, USA, 2007 (76)	prospective observational study	y	y	y	y	y	y	y	y	y	y	y			good
Rhodes A, Martin S, Wolters P, et al, USA, 2020, (77)	cross-sectional study	y	y	y	y	n	n	y	y						good
Ballas SK et al, USA, 2006 (78)	randomized clinical trial	y	y	y	y	y	y	y	y	y	y	y	y	y	good
Badawy SM et al, USA, 2017 (79)	cross-sectional study	y	n	y	y	n	U	y	y						fair
Badawy SM et al, USA, 2017 (80)	cross-sectional study	y	n	y	y	n	U	y	y						fair
Badawy SM et al, USA, 2017 (81)	cross-sectional study	y	n	n	y	n	U	y	y						fair
Badawy SM et al, USA, 2018 (82)	cross-sectional study	y	n	y	y	y	y	y	y						good
Badawy SM et al, USA, 2018 (83)	cross-sectional study	y	n	y	y	y	y	n	y						good
Maxwell SL et al, USA, 2019 (84)	cross sectional study	n	U	y	y	y	y	y	y						good

Table 2 - risk of bias assessment of thalassemia studies
Legend: y: yes, n: no, NA: not applicable, U: unclear.

Author, Country, Year (reference)	Study design	1	2	3	4	5	6	7	8	9	10	11	12	13	Rating
Arian M et al, 2018 (11)	systematic review	y	y	y	y	y	y	U	y	y	y	y			good
Dimitroglou Y et al, Greece, 2020 (12)	cross-sectional study	y	y	y	y	y	y	y	y						good
Etemad, K. et al, Iran, 2021 (13)	cross-sectional study	y	y	y	y	y	y	y	y						good
Klaassen RJ et al, USA, 2014 (28)	cross-sectional study	y	y	y	y	y	y	y	y						good
Haghpanah S et al, Iran, 2013 (85)	cross-sectional study	y	y	y	y	n	U	y	y						good
Jafari H et al, iran, 2008 (86)	cross-sectional study	y	y	y	y	y	y	y	y						good
Sharifi F et al, Iran, 2021 (87)	cross-sectional study	U	y	y	y	y	y	y	y						good
Musallam KM et al, Lebanon, 2011 (88)	cross-sectional study	y	y	y	y	n	U	y	y						good
Safizadeh H et al, Iran, 2012 (89)	cross-sectional study	n	U	y	y	n	n	y	y						fair
Adam S et al, Saudi Arabia, 2019 (90)	cross-sectional study	y	y	y	y	y	y	y	y						good
Javanbakht M et al, Iran, 2009 (91)	cross-sectional study	y	y	y	y	y	y	y	y						good
Yilmaz, Y. et al, Turkey, 2017 (92)	cross-sectional study	y	y	y	y	y	y	y	y						good
Töret E et al, Turkey, 2018 (93)	cross-sectional study	n	n	y	y	n	U	y	y						fair
Yasmeen H et al, Pakistan, 2018 (94)	cross-sectional study	y	y	y	y	y	y	y	y						good
Dehkordi A et al, Iran, 2020 (95)	randomized clinical trial	y	y	y	U	U	U	y	y	y	y	y	y	y	good
Adib-Hajbaghery M et al, Iran, 2017 (96)	cross-sectional study	n	n	y	y	y	y	y	y						good
Al-Moshary M et al, Pakistan, 2019 (97)	cross-sectional study	n	n	y	y	n	n	y	y						fair
Adib-Hajbaghery M et al, Iran, 2015 (98)	cross-sectional study	n	n	y	y	y	y	y	y						good
Azarkeivan A et al, Iran, 2009 (99)	cross-sectional study	y	y	y	y	y	y	y	y						good
Hajibeigi B et al, Iran, 2009 (100)	cross-sectional study	n	n	y	y	y	y	y	y						good
Yengil E et al, Turkey, 2014 (101)	cross-sectional study	y	y	y	y	y	y	y	y						good
Khani H et al, Iran, 2012 (102)	cross-sectional study	n	n	y	y	y	y	y	y						good

Siddiqui SH et al, Pakistan, 2014 (103)	cross-sectional study	y	y	y	y	y	y	y	y						good
Amoudi AS et al, Saudi Arabia, 2014 (104)	cross-sectional study	y	y	y	y	y	y	y	y						good
Paramore C et al, Itali, UK, USA, 2021 (105)	prospective observational study	NA	y	y	y	y	y	y	y	y	y	y			good
Payne KA et al, USA, 2007 (106)	cross-sectional study	y	y	y	y	U	U	y	y						good
Sobota A et al, USA, Canada, UK, 2011 (107)	cross-sectional study	y	n	y	y	y	y	y	y						good
Gollo G et al, Italy, 2009 (108)	cross-sectional study	y	y	y	y	y	y	y	y						good
Goulas V et al, Greece, 2012 (109)	prospective observational study	y	y	y	n	n	y	y	U	U	U	y			fair
Goulas V et al, Greece, 2021 (110)	cross-sectional study	n	n	y	y	y	y	y	y						good
La Nasa G et al, Italy, 2013 (111)	cross-sectional study	n	y	y	y	y	y	y	y						good
Klonizakis P et al, Greece, 2017 (112)	cross-sectional study	y	y	y	y	y	y	y	y						good
Messina G et al, Italy, 2008 (113)	cross-sectional study	y	y	y	y	y	y	y	y						good
Lam JCM et al, Singapore, 2021 (114)	cross-sectional study	n	y	y	y	n	U	y	y						fair
Sharma S et al, India, 2017 (115)	cross-sectional study	y	y	y	y	y	y	y	y						good
Dahlui M et al, Malaysia, 2009 (116)	cross-sectional study	y	y	y	y	y	y	y	y						good
