# Supplementary material for: Health-related quality of life in hemoglobinopathies: A systematic review from a global perspective
Source: Front Pediatr. 2022 Aug 25;10:886674. doi: 10.3389/fped.2022.886674 (PMC9452907; doi:10.3389/fped.2022.886674)
Supplement: Supplementary file 3 [file Data_Sheet_3.PDF]

Supplementary materials 3

Table 1.1 Characteristics of SCD selected articles (n=66)

Geographic area	Number of articles	Questionnaire adopted							Population					
		Generic			Disease-specific		Other	Articles in which was mentioned translation in local language	Children ≤ 14 yo	Adolescents 14-18 yo	Adults >18 yo	Mixed		
		SF-36	PROMIS	PedsQL	SCSES	ASCQ-Me						children and adolescents	adolescents and adults	children, adolescents and adults
Middle East	4/66 (6%)	3/4 (75%)	-	1 (SCD Module) (25%)	-	-	-	4/4 (persian, arabic) (100%)	1	-	1	-	2	-
Latin America and Caribbean	14/66 (21%)	12/14 (86%)	-	3/14 (2 SCD Module) In 1 case combined with SF-36 (21%)	-	-	WHOQOL-Bref (combined with SF-36)	7/12 (portuguese) (58%)	2	-	7	2	3	-
Africa	4/66 (6%)	2/4 (50%)	-	1/4 (25%)	1/4 (25%)	-	-	2/4 (portuguese, english) (50%)	1	-	2	-	-	1
USA	42/66 (64%)	8/42 (19%)	19/42 (45%)	10/42 (SCD Module, 1 combined with PROMIS) (24%)	1/42 (2%)	7/42 (2 combined with PROMIS) (17%)	-	-	2	-	16	19	4	1
Europe	1/66 (2%)	1	-	-	-	1 (combined with SF-36)	-	-	-	-	1	-	-	-
Generic review	1	-	-	-	-	-	-	-	-	-	-	-	-	-
TOTAL	66	26/66 (39%)	19/66 (29%)	15/66 (23%)	2/66 (3%)	8/66 (12%)	1/66 (1%)	13/66 (20%)	6/66 (9%)	0/66	27/66 (41%)	21/66 (32%)	9/66 (14%)	2/66 (3%)

Table 1.2 - HRQOL in SCD - summary of the included studies

Author, Country, Year (reference)	Study design	Median age	Number of participants	QoL questionnaire	Questionnaire language	Topic/Results
Bulgin D et al, USA, 2019 (7)	cross-sectional study	38.5 ± 13.7	20	ASCQ-Me	english	There was good participation in completing the ASCQ-Me and J-SAT questionnaires, low refusal rates, and subjects completed the instruments and items without difficulty
Singh SA et al, USA, 2020, (14)	review	-	-	PROMIS, ASCQ-Me, PedsQL, SF-36	-	PRO data provide a patient-centric disease perspective that can lead to improved quality of care and treatment adherence by shared decision-making
Singh A et al, USA, 2019, (17)	cross-sectional study	10 ± 4	117	PROMIS	english	The new domains of physical stress experience, strength impact, pain behavior, and pain quality were found to be valid for children with SCD
Singh A et al, USA, 2019, (18)	cross-sectional study	11.5 ± 2.8	117	PROMIS	english	The study identified T-scores for PROMIS pain domains that facilitate clinical interpretation and provide necessary information for PROMIS users in a clinical setting
Singh A et al, USA, 2020, (19)	prospective cohort study	10.7 ± 3.6	67	PROMIS	english	PROMIS domains related to pain and physical functioning detect changes in the health status in vaso-occlusive crises, analyse pain in time and response to treatment
Panepinto JA et al, USA, 2012, (22)	qualitative study	2-18	103	PedsQL SCD	english	Qualitative methods involving pediatric patients and parents in the item development process support the content validity for the PedsQL SCD Module
Panepinto JA et al, USA, 2013, (23)	qualitative study	2-18	321	PedsQL SCD	english	In conjunction with the PedsQL, the PedsQL SCD Module facilitate the understanding of the health and well-being of children with SCD
Beverung LM et al, USA, 2015 (24)	cross-sectional study	11.47 ± 3.84	251	PedsQL SCD, PedsQL	english	Scores of 60 or below on the PedsQL SCD Pain and Hurt and Pain Impact scales are associated with severe disease and meet requirements for impaired functioning on the PedsQL Generic Core Scales
Edwards R et al, USA, 2000 (25)	cross-sectional study	38.7 ± 12.8	83	SCSES	english	The SCSES have good internal consistency, discriminant validity, and convergent and predictive validity
Keller SD et al, USA, 2014 (26)	cross-sectional study	>18	556	ASCQ-Me	english	The study identified three subfactors within physical impact: sleep, pain and stiffness impacts. Analysis of the resulting six item sets (sleep, pain, stiffness, cognitive, emotional and social impacts of SCD) supported their essential unidimensionality.
Treadwell M. J. et al, USA, 2014 (27)	comprehensive literature review	-	86 articles	ASCQ-Me	english	The effects of pain on QOL are predominating, interwoven with emotional distress, quality of care, and stigmatization.
Ahmed AE et al, Saudi Arabia, 2015 (30)	multicenter, cross-sectional study	28.8 ± 9.1	629	SF-36	arabic	Poor education, fever, skin redness and swelling are negatively associated with specific components of SF-36. A history of blood transfusion decreases QoL. Regular exercise tends to improve QoL
Khaled A et al, Saudi Arabia, 2021 (31)	cross-sectional study	25 ± 7.85	107	SF-36	arabic	Exercise and family support affect the physical health, mental health and vitality scores positively, while swelling and fever have a negative effect on physical and mental health. More educated patients demonstrate significantly better mental health than less educated patients
Ahmadi M et al, Iran, 2015 (32)	one-group before and after	25.84 ± 7.23	69	SF-36	persian	Self-management interventions increase the total QoL score

Pandarakutty S et al, Oman, 2019, (33)	quasi-experimental nonequivalent control group	8-12	30	PedsQL SCD	arabic	After nurse led intervention, the HRQOL score significantly improve
Asnani MR et al, Jamaica, 2009 (34)	cross-sectional study	cohort vs main, 27.6±2.5 vs 34.7±12.2	552	SF-36	not mentioned	There is evidence of the validity of the SF-36 in Jamaicans with SCD
Asnani MR et al, Jamaica, 2007 (35)	cross-sectional study	adults	-	SF-36	not mentioned	Within Jamaican samples of patients, the SF-36 has a component structure which is quite distinct from that initially proposed by its creators
Asnani MR, Jamaica, 2009 (36)	cross-sectional study	1.3 ± 9.6	491	WHOQOL-Bref	U.K.version interviewer-administered	the WHOQOL-Bref instrument has good psychometric properties in determining QOL
Menezes AS de O d. P et al, Brazil, 2013 (37)	cross-sectional study	5-18	100	PedsQL, SF-36	portuguese	Lower scores were obtained by patients and patient's parents than the control group
Rodrigues CF de A et al, Brazil, 2021, (38)	cross-sectional study	>14 pts	113	SF-36	portuguese	Patients diagnosed with SCD who reported perception of prejudice had statistically significant worse QoL, revealing the negative impact, that might lead to sadness and social isolation
Oliveira, C. D. L. et al, Brazil, 2019 (39)	cross-sectional study	10 (8-12)	412	PedsQL SCD	portuguese	QoL of this patient population varied according the health center even adjusted by socio-demographics characteristics
Osborne JC et al, USA, 2020, (40)	cross-sectional study	13.6 ± 2.4	28	PedsQL SCD	Spanish	The Spanish version of the PedsQL SCD Module reduces a barrier to measurement of disease-specific QOL of Spanish-speaking children and their parents.
Blake A et al, Jamaica, 2020 (41)	cross-sectional study	15.2 ± 1.5	102	PedsQL SCD	not free access	Parents and adolescents exhibit fair agreement in assessment of the adolescent's overall HRQOL but differ on subjective domains. Agreement varies by sex of the affected teen: girls' HRQOL is generally overestimated by the parental proxy
Asnani MR et al, Jamaica, 2008 (42)	observational, analytical study	urban vs rural, 35.8 ± 12.5 vs 32.8 ± 11.2	166	SF-36	not mentioned	The rural sample assessed their QOL as higher than the urban sample
Ohara DG et al, Brazil, 2012 (43)	cross-sectional study	31.77 ± 1.87	27	SF-36	portuguese	Body pain is associated with race and education and all pain areas are associated with the physical components of the SF-36. Pain is significantly associated with vitality and mental health components of the SF-36.
Goncalves CEA et al, Brazil, 2019 (44)	cross-sectional study	26 ± 6	33	SF-37	not mentioned	Adults with SCA have muscle dysfunction, especially with regard to endurance of the knee flexor muscles. There is a significant association between muscle function and HRQoL. The use of hydroxyurea is associated with better HRQoL and less muscle dysfunction.
dos Santos JP et al, Brazil, 2013 (45)	cross-sectional study	31.9 ± 12.67	32	SF-36	portuguese	The disease interferes in the working capacity of individuals, who mostly have low incomes and impaired access to healthcare services and significantly impacts on their quality of life

Almeida CHS de, Brazil, 2021 (46)	one-group before and after	31.8 ± 14.5	40	SF-36	not mentioned	Therapist-oriented home rehabilitation can increase functional capacity, muscle strength and QoL in adults
Tinti G et al, Brasil, 2010, (47)	case report before and after	32	1	SF-36	portuguese	Aquatic rehabilitation can be used to improve clinical conditions of patients
Gibson RC et al, Jamaica, 2013 (48)	cross-sectional study	36.4 ± 12.2	143	SF-36	not mentioned	The perception of life as being determined by oneself behavior, a concept known as internal locus of control, is associated with high HRQOL, while the perception of life as being determined by external factors, known as external LOC, is associated with depression
Andong AM et al, Cameroon, 2017 (49)	cross-sectional study	16.0 (9.0–24.0)	175	SF-36	not mentioned	The prevalence of chronic complications is higher than in most other SCD populations worldwide and are the main drivers of low QOL
Issa F et al, Mozambique, 2020 (50)	cross-sectional study	2-14 years	14	PedsQL	portuguese	In a population of Mozambican pediatric patients and caregivers it has been found lower quality of life in emotional and communication domains, than in pain-related ones
Aloba O et al, Nigeria, 2020 (51)	correlational analysis between variables	25.38 ± 6.73	123	SCSES	not mentioned	Hopelessness is influenced by Hb concentration and psychological variables (resilience and depression)
Ojelabi, AO et al, Nigeria, 2019 (52)	cross-sectional study	27.9 ± 6.95	200	SF-36	not mentioned	Utility score is low indicating a substantial impact of the disease on HRQOL of patients and the value they place on their health state due to the limitations they experienced
Lee S et al, USA, 2020 (53)	systematic review of literature	>18	22 studies	SF-36	english	Disease burden measured by PROs and economic burden of SCD on adults in the US are substantial despite the availability of approved SCD treatments during 2007-2018
Knisely MR et al, USA, 2020 (54)	cross-sectional study	29.2 ± 7.2	2201	ASCQ-Me, PROMIS	english	In multivariate models, patient reported outcomes were significantly associated with one another
Dampier C et al, USA, 2016 (55)	cross-sectional study	12.5 ± 3.1	121	PROMIS	english	Pain interference and fatigue domain scores are elevated at baseline, increase substantially during hospitalization, and largely return to baseline by the recovery period. The depressive symptoms, anger, and anxiety domain scores display a less pronounced elevation during hospitalizations and a slower return to baseline levels
Dampier C et al, USA, 2016 (56)	cross-sectional study	12.5 ± 2.8	235	PROMIS	english	PROMIS pediatric measures are feasible in a research setting and identify expected differences in known group comparisons in a sample of SCD children
Hildenbrand AK et al, USA, 2019 (57)	cross-sectional study	20.81 ± 1.73	45	PROMIS, PedsQL	english	Findings provide preliminary evidence of validity and reliability of the PROMIS Pain Interference and Fatigue short forms among emerging adults with SCD
Keller S et al, USA, 2017 (58)	cross-sectional study	>18	490	ASCQ-Me	english	Study results support the validity of both PROMIS and ASCQ-Me measures for use in adults with SCD
Cooper, O. et al, UK, 2019 (59)	cross-sectional study	36.1 ±12.5	173	ASCQ-Me, SF-36	english	The analysis of the sample shows evidence of both validity and reliability of the ASCQ-Me for use in the UK SCD population
Curtis S et al, 2017 (60)	systematic review of literature	adults and children, separately	2 articles	PROMIS	english	Pain domains from both PROMIS measures and PedsQL SCD module are valid, reliable, and responsive to changes in acute pain in children with SCD. Though, to date neither has been shown to be responsive to changes in chronic pain over time

McClish DK et al, USA, 2005 (61)	cross-sectional study	33 (16 - 64)	308	SF-36	english	Patients experience health related quality of life worse than the general population, and in general, their scores were most similar to patients undergoing hemodialysis
Esham KS et al, USA, 2020 (62)	cross-sectional study	30 ± 9.1	42	PROMIS, ASCQ-Me	english	Among adults hospitalized for vaso-occlusive crisis nearly all PROMIS and ASCQ-Me scores are worse than population norms. Scores improve after discharge, driven principally by improvements in pain
Dampier C et al, USA, 2011 (63)	cross-sectional study	31.4 ± 11.8	1046	SF-36	english	The occurrence of specific complications diminished SF-36 scale scores: VOC, asthma and avascular necrosis
Rizio AA et al, USA, 2020, (64)	cross-sectional study	34.37 ± 10.25	303	ASCQ-Me	english	patients with SCD who had more frequent or severe VOCs experienced deficits in multiple domains of HRQoL and work productivity
Brandow AM et al, USA, 2016 (65)	secondary analysis of prospectively collected data from the randomized controlled MAGiC trial	13.6 ± 4.7	204	PedsQL SCD	english	Earlier initiation and higher doses of oral opioids administered at the emergency department for VOC is strongly associated with shorter length of stay and improved HRQOL at hospital discharge
Karafin MS et al, USA, 2018 (66)	cross-sectional study	30 ± 5	99	SF-36	english	Higher daily opioid dose is associated with chronic pain. Among those with chronic pain, opioid dose ≥ 90 MME is associated with worse HRQOL
Bakshi N et al, USA, 2018 (67)	randomized clinical trial	35	47	PROMIS	english	The presence of pain on 3 or more days a week is associated with worse patient-reported pain interference and anxiety
Badawy SM et al, USA, 2018 (68)	cross-sectional study	13.5	34	PROMIS	english	Older and female participants have worse QOL scores, and males report higher hydroxyurea adherence. Participants with chronic pain report significant impairment in different QOL domains and have increased healthcare utilization
Connolly ME et al, USA, 2019 (69)	institutional review board–approved study	10.4 ± 2.9	89	PedsQL-SCD Module	english	Patients with persistent pain demonstrate poorer working memory and processing speed
Román ME et al, Winsconsin, 2020, (70)	cross-sectional study	15 (14-16.5)	12	PedsQL SCD	english	Adolescents with SCD and neuropathic pain have poor HRQL even in their baseline state of health
Ameringer S et al, Virginia, 2014, (71)	descriptive, correlational study	22.5 ± 4.1	60	PROMIS	english	Fatigue is common and intereferes with daily activities such as school, work and exercise
Sogutlu A et al, Virginia, 2011, (72)	cross-sectional study	34.4 ± 11.4	230	SF-35	english	High somatic symptom burdenwas 1.5 to 2 times more prevalent in SCD patients than in primary care. High SSB predicts more non-crisis pain and healthcare utilization for pain, and is associated with depression, anxiety, and poorer HRQOL

Levenson, J. L. et al, USA, 2008 (73)	longitudinal, epidemiologic study	34	232	SF-37	english	Depression and anxiety predict more daily pain and poorer physical and mental quality-of-life in adults with SCD, and accounted for more of the variance in all domains of quality-of-life than hemoglobin type
Miller M et al, USA, 2021 (74)	cross-sectional study	13.02 ± 0.47	51	PROMIS	english	Use of maladaptive emotion regulation strategies is associated with increased symptoms of depression, anxiety and pain interference
Bakshi N et al, USA, 2018 (75)	cross-sectional study	15	33	PedsQL, PedsQL SCD	english	Pain catastrophizing is inversely correlated with HRQoL
Citero V de A et al, USA, 2007 (76)	prospective cohort study	34 ± 11.4 years	220	SF-36	english	Adults with SCD have a higher mean catastrophizing score than found in studies of other chronic pain conditions that are not lifelong and life-threatening
Rhodes A, Martin S, Wolters P, et al, USA, 2020, (77)	cross-sectional study	32 ± 7.79	62	PROMIS	english	The relationship between sleep and executive skills must be considered in the context of anxiety among adults with SCD
Ballas SK et al, USA, 2006 (78)	randomized, double-blind, placebo-controlled trial	18–29 (51%), 30–39 (39%), 40–49 (9%), >=50 (1%)	299	SF-36	english	Treatment with hydroxyurea improves some aspects of QOL
Badawy SM et al, USA, 2017 (79)	cross-sectional study	13.5	34	PROMIS	english	SCD patients with low hydroxyurea adherence and/or low HbF or MCV levels have worse HRQOL scores, particularly fatigue
Badawy SM et al, USA, 2017 (80)	cross-sectional study	13.5	34	PROMIS	english	Patients with fewer barriers to hydroxyurea adherence are more likely to have higher adherence rates and better HRQOL scores
Badawy SM et al, USA, 2017 (81)	cross-sectional study	13.5	34	PROMIS	english	Participants with higher hydroxyurea adherence perceive more hydroxyurea benefits and have better emotional response
Badawy SM et al, USA, 2018 (82)	cross-sectional study	13.5	34	PROMIS	english	Beliefs about hydroxyurea correlate with HRQOL scores and adherence levels
Badawy SM et al, USA, 2018 (83)	cross-sectional study	13.5	34	PROMIS	english	Increased healthcare utilization in youth with SCD is associated with low adherence to hydroxyurea and worse HRQOL scores
Maxwell SL et al, USA, 2019 (84)	retrospective chart review study	14.04 ± 3.09	67	PedsQL SC	english	children in the CRCT group had significantly higher self-reported HRQL ratings for domains related to pain and pain-related functioning compared with children with similar and milder disease risk

Table 2.1 Characteristics of thalassemia selected articles (n=36)

Geographic area	Number of articles	Questionnaire adopted					Population					
		Generic		Disease-specific	Other	Articles in which was mentioned translation in local language	Children ≤ 14 yo	Adolescents 14-18 yo	Adults > 18 yo	Mixed		
										Children and adolescents	Adolescents and adults	Children, adolescents and adults
		SF-36	PedsQL	TranQoL								
Middle East	21/36 (58,3%)	17/21 (81%)	-	4/21 (19%)	6/21 (28,6%)	11/21 (52,3%)	-	-	2/21 (9,5%)	-	-	19/21 (90,5%)
USA and Canada	4/36 (11,1%)	3/4 (75%)	1/4 (25%)	2/4 (50%)	3/4 (75%)	-	-	-	-	-	1/4 (25%)	3/4 (75%)
Europe	9*/36 (25%)	8/9 (89%)	-	1/9 (11%)	4/9 (44,4%)	6/9 (66,7%)	-	-	6/9 (66,7%)	-	1**/9 (11%)	2***/9 (22,2%)
South East Asia	3/36 (8,3%)	2/3 (66.7%)	1/3 (33,3%)	1/3 (33,3%)	1/3 (33,3%)	-	-	-	-	1/3 (33,3%)	-	⅔ (66,7%)
Worldwide	1/36 (2,8%)	1	-	-	-	-						
TOTAL	36	31/36 (86,1%)	2/36 (5,6%)	6/36 (16,7%)	13/36 (36,1%)	17/36 (47,2%)	-	-	8/36 (22,2%)	1/36 (2,8%)	1/36 (2,8%)	25/36 (69,4%)

* 2 studies are conducted in Europe, USA and Canada
** study conducted in Europe, USA and Canada
*** 1 study conducted in Europe, USA and Canada

Table 2.2 - HRQOL in thalassemia - summary of the included studies

Author, Country, Year (reference)	Study design	Median age	Number of participants	HRQOL questionnaire	Questionnaire language	Topic/Results
Arian M et al, 2018 (11)	systematic review	21.79	2961	SF-36	not mentioned	Measuring HRQoL should be considered as an essential part of the overall assessment of health status of BTM patients, which would provide valuable clues for improving the management of disease and making decisions on the treatment
Dimitroglou Y et al, Greece, 2020 (12)	cross-sectional study	> 18	73	SF-36	not mentioned	HRQOL is negatively affected by the severity of heart failure and its stage in beta-thalassemia patients
Etemad, K. et al, Iran, 2021 (13)	cross-sectional study	children and adults	1240	TranQol, MSPSS	not mentioned	Adults had higher HRQOL score than children under 15 years old. The multivariable linear regression analysis showed that the age, gender, age of blood transfusion initiation, Hb level, number of underlying diseases, and social support level by family and community significantly impact HRQOL
Klaassen RJ et al, USA, 2014 (28)	cross-sectional study	20.7	106	TranQoL, PedsQL, SF-36, HUI3	not mentioned	The TranQol is a valid and reliable instrument of assessing HRQOL in thalassaemia major patients
Haghpanah S et al, Iran, 2013 (85)	cross-sectional study	19.52 ± 4.3	101	SF-36	Persian	Lower income, poor compliance with iron-chelating therapy and presence of comorbidities were significantly correlated with lower SF-36 scores. These factors were also found to be determinants of worse SF-36 scores in multivariate analysis
Jafari H et al, iran, 2008 (86)	cross-sectional study	19.81 ± 4.07	200	SF-36	Persian	The Persian version of SF-36 questionnaire is a reliable and valid instrument of thalassemia major HRQOL assessment
Sharifi F et al, Iran, 2021 (87)	cross-sectional study	22.45	324	New questionnaire (20 questions in 5 subscale) summarizing SF-36	not mentioned	The Rasch model as a precise tool helped us develop a new reliable and valid questionnaire to understand individuals' HRQOL
Musallam KM et al, Lebanon, 2011 (88)	cross-sectional study	>18	80	RAND SF-36	Arabic	Patients with transfusion-independent thalassemia intermedia have lower HRQOL compared to thalassemia major patients
Safizadeh H et al, Iran, 2012 (89)	cross-sectional study	22.95 ± 4.82	308	SF-36	not mentioned	HRQOL of patients with thalassemia major was better than that of patients with thalassemia intermedia in Physical Functioning and Role limitation-Emotional
Adam S et al, Saudi Arabia, 2019 (90)	cross-sectional study	22.9 ± 11.7	105	SF-36	arabic	Higher pretransfusion Hb and younger age were associated with better mental HRQoL outcomes. Professionals reported better physical HRQoL outcomes, compared to non-professionals
Javanbakht M et al, Iran, 2009 (91)	cross-sectional study	20	196	SF-36	not mentioned	HSCT in beta thalassemia patients improves HRQoL in physical and mental aspects, HRQOL was higher in comparison with HRQOL of patients treated with iron chelation therapy

Yilmaz Y et al, Turkey, 2017 (92)	cross-sectional study	Controls group: 26.6; case group: 28	84	SF-36, Snap & Sniff wands	not mentioned	Reduced olfactory function in Transfusion Dependent Thalassemia patient decreases HRQOL.
Töret E et al, Turkey, 2018 (93)	cross-sectional study	0 - 39	107	SF-36, BDI	not mentioned	Beta thalassemia patients have low SF-36 subscale scores. Patients younger than 20 years had worse mental health scores and patients older than 20 years had worse physical role scores. High levels of depression were found
Yasmeen H et al, Pakistan, 2018 (94)	cross- sectional study	5 - 25	174	TranQoL	not mentioned	Family history, death , monetary issues , income , education, pre-transfusion Hb and transfusions frequency are associated with lower HRQOL
Dehkordi A et al, Iran, 2020 (95)	randomized clinical trial	> 18	40	SF-36	not mentioned	Exercise in water improves HRQOL
Adib-Hajbaghery M et al, Iran, 2017 (96)	cross-sectional study	25.74 ± 5.72	154	TranQoL	persian	The Persian translation of the TranQoL questionnaire is highly reliable and valid.
Al-Moshary M et al, Pakistan, 2019 (97)	cross-sectional study	1.54 ± 3.6	431	TranQoL	not mentioned	HRQOL is low in thalassemia intermedia patients
Adib-Hajbaghery M et al, Iran, 2015, (98)	cross-sectional study	> 12	173	SF-36, DAS-21	not mentioned	Beta thalassemia major is related to low HRQOL. A majority of the β-TM patients also suffered from mild to severe depression, anxiety and stress
Azarkeivan A et al, Iran, 2009 (99)	cross-sectional study	>18	172	SF-36	not mentioned	Depression is associated with both poor physical and mental HRQOL among patients with major/intermedia beta thalassemia. Somatic comorbidities and anxiety are associated with poor physical and mental HRQOL
Hajibeigi B et al, Iran, 2009 (100)	cross-sectional study	25 ± 6	292	SF-36, HADS, PSQI	Persian	Mental and physical quality of life scores were predicted by symptoms of depression and somatic comorbidities. Total sleep quality was predicted by anxiety symptoms and somatic comorbidities
Yengil E et al, Turkey, 2014 (101)	cross- sectional study	patients: 17.42 ± 4.65; caregivers: 36.25 ± 9.50	151 (88 patients, 63 caregivers)	SF- 36, BDI, BAI	not mentioned	Physical health component summary score is influenced by depression scores. Mental health component score is influenced by depression scores. Physical functioning and general health scores are lower in patients compared to their caregivers
Khani H et al, Iran, 2012 (102)	cross-sectional study	22.33	687	SF-36, SCL-90-R, LSI	not mentioned	The 47.9% of participants showed an excellent level of physical functioning in SF-36. SCL-90-R and LSI scores suggest that thalassemia major patients are at risk of psychiatric symptoms.
Siddiqui SH et al, Pakistan, 2014 (103)	cross-sectional study	6 - 21 years old, mean age 10.5 years old	101	SF-36	not mentioned	Physical impairments, social stresses, financial burdens and problems with their education and career decrease HRQOL - psychological functioning
Amoudi AS et al, Saudi Arabia, 2014 (104)	cross-sectional study	26.02 ± 5.56	48	SF-36	not mentioned	Psychological functioning shows low score, social functioning scores were low in females and non-Saudis
Paramore C et al, Itali, UK, USA, 2021 (105)	prospective observational study	>18	85	TranQoL, BFI, BPI-SF	not mentioned	The patient- and caregiver-reported burden is high, strongly influenced by disease-management time, fatigue and pain symptoms, and impairment to quality of life.

Payne KA et al, USA, 2007 (106)	cross-sectional study	> 6	49	SF-36, CHQ	not mentioned	HRQOL, measured by the SF-36, and treatment with iron chelation therapy satisfaction appear compromised
Sobota A et al, USA, Canada, UK, 2011 (107)	cross-sectional study	>14	263	SF-36	not mentioned	HRQOL lower than US norms with the greatest effect in general health and the physical domains; in the UK older age, greater number of side effects and number of complications were the major factors associated with lower HRQOL
Gollo G et al, Italy, 2009 (108)	cross-sectional study	> 18	43	SF-36	italian	HRQOL assessed by SF-36 improves significantly from 2001 to 2009
Goulas V et al, Greece, 2012 (109)	prospective observational study	> 18	135	SF-36	Greek	HRQOL of beta-thalassemia patients receiving chelation therapy is dependent on the type of iron chelation treatment they receive
Goulas V et al, Greece, 2021 (110)	cross-sectional study	41	131	SF-36, SICT	Greek	No differences in HRQOL were noted between patients receiving parenteral versus oral ICT
La Nasa G et al, Italy, 2013 (111)	cross-sectional study	34	130	SF-36, FACT-BMT	Italian	Clinical meaningful differences were only found for general health. Mental health, education level, employment status, marital status, living arrangements, and birth rate were compatible with normal living patterns. The development of GVHD and older age at transplantation were important impairing factors. Patients receiving conventional treatment of β-thalassemia revealed poorer outcomes compared with the cohort of transplanted patients
Klonizakis P et al, Greece, 2017 (112)	cross sectional study	32.1	94	TranQol, SF-36	Greek	This study shows a strong correlation between HRQOL investigation through TranQol and SF-36v2.
Messina G et al, Italy, 2008 (113)	cross sectional study	45	147	SF-36, WCQ, SCL-90-R	Italian	Evaluation of mean values of symptomatological dimensions in these patients showed a personality characterized by somatization, depression and obsessive-compulsive traits. SF-36 showed low scores in emotional and social functioning
Lam JCM et al, Singapore, 2021 (114)	cross sectional study	21	100	TranQOL	not mentioned	The cohort of older thalassaemia patients experienced significantly higher rates of cardiac iron loading, endocrine complications and lower TranQOL scores compared to younger age cohorts
Sharma S et al, India, 2017 (115)	cross sectional study	2 - 18	155 (75 cases, 80 controls)	SF-36, PedsQL	not mentioned	HRQOL of children with beta-thalassemia major was significantly The lowest scores were obtained in the domain of school functioning. Social functioning was not significantly affected
Dahlui M et al, Malaysia, 2009 (116)	cross sectional study	not mentioned	112	SF-36, MOS	Bahasa Malaysia	Optimum desferrioxamine usage reduces iron overload complications and provides a better quality of life
